# Barriers and facilitators of early postpartum modern contraceptive method uptake in Dessie and Kombolcha City zones, northeast Ethiopia: Conventional content analysis qualitative study

**DOI:** 10.1371/journal.pone.0305971

**Published:** 2024-07-17

**Authors:** Niguss Cherie, Muluemebet Abera Wordofa, Gurmesa Tura Debelew

**Affiliations:** 1 Population and Family Health Department, Institute of Health, Faculty of Public Health, Jimma University, Jimma, Ethiopia; 2 Reproductive and Family Health Department, School of Public Health, College of Medicine and Health Sciences, Wollo University, Dessie, Ethiopia; Arba Minch University, ETHIOPIA

## Abstract

**Background:**

Despite progress in access to family planning services in many sub-Saharan African countries in recent decades, advances in effective early postpartum contraceptive adoption remain low, and the unmet need for early postpartum contraceptives is high. In Ethiopia, early postpartum modern contraceptive method uptake is still unacceptably low. The barriers/challenges have not yet been sufficiently explored. A deep and detailed understanding of the contextualized barriers and challenges in the adoption of early postpartum contraceptive methods is crucial in developing future locally-appropriate interventions.

**Objectives:**

This study aimed to explore barriers/challenges to the uptake of early postpartum modern contraceptive methods after childbirth in Dessie and Kombolcha zones, in northeast Ethiopia.

**Methods:**

Aconventional content analysis qualitative study was deployed in Dessie and Kombolcha town zones, northeast Ethiopia using a theoretical purposive sampling technique. A total of 57 study subjects were participated. The sample size was determined using the rule of information saturation through 7 key informant interviews, 6 in-depth interviews, and 5 focused-group discussions with 8–10 participants each. Data were collected using an unstructured interview guide and recorded using a digital audio recorder and field notes. The trustworthiness of the study was assured using different techniques. The collected data were transcribed and translated from native language to English. Atlas-ti version7 software was used to facilitate conventional content qualitative data analysis approach. Open coding, categories, subthemes, and overreaching themes were developed, and a conceptual model of barriers was organized through network analysis.

**Result:**

Barriers to uptake of early postpartum modern contraception quoted by study participants and themed were related knowledge, attitude, family-community, health facility, contraceptive method, cultural, religious, fertility desire, gender issues, and misconceptions. The sub-themes of knowledge-related barriers that emerged were lack of awareness of the time to take birth control methods, not knowing the time pregnancy is likely after childbirth, and not being committed to taking contraceptives early enough after childbirth. Moreover, beliefs that modern contraceptives cause breast milk to dry up and perceived low fecundability after childbirth were indicated as attitude barriers. Health facility barriers were lack of reminders and follow-up mechanisms, sporadic service delivery and opening time, long waiting time, and card withdrawal process and providers’ approach. Social stigma, child sex preference, and religious restrictions against contraceptive use were community barriers.

**Conclusion:**

Generally individual, facility-based, method-related, misconceptions, societal, and cultural barriers were identified as hindrances to the uptake of early postpartum modern contraceptive methods. There is a need for health-seeking behavioral interventions, innovative contraceptive methods, and facility-level interventions to overcome each identified barrier.

## Introduction

Early Post-Partum (EPP) contraceptive method uptake is the adoption of modern contraceptive methods within the first 6 weeks following childbirth to prevent closely spaced and unintended pregnancies [1, 2]. Early postpartum contraception and delaying the next pregnancy for at least 24–36 months before attempting conception is recommended to reduce the risk of adverse maternal, perinatal, and infant outcomes [3]. Pregnancy occurring within six months of the last delivery holds a 7.5 times increased risk for induced abortion, a 3.3-fold increase in miscarriage, and a 1.6-fold increased risk of stillbirth [4]. The promotion of early postpartum contraception in countries with high birth rates has the potential to avert 32% of all maternal deaths and nearly 10% of childhood deaths [5].

During the early postpartum period, contraceptive methods prevent about 30% of maternal mortality and 10% of child mortality if couples space their pregnancies for more than 2 years apart [6]. Conversely, closely spaced pregnancies within the first year postpartum increase the risks of preterm birth, low birth weight, small-for-gestational-age babies, and maternal death [7]. The timing of the return to fertility after childbirth is variable and unpredictable. Women can get pregnant before the return of menstruation [8]. Though the World Health Organization (WHO) recommends that pregnancies be spaced by at least 24 months, nearly half (47%) of postpartum women have short birth to pregnancy intervals in Ethiopia [9].

Despite progress in access to family planning services in many sub-Saharan African countries in recent decades, advances in effective early postpartum contraceptive use remain low and the unmet need for postpartum contraceptives remains unacceptably high [10]. According to estimates derived from Demographic Health Survey (DHS) data analysis from 57 countries during the 2005–2019 period unmet need for early postpartum contraceptive method was 62%. The unmet need for contraceptive methods right after birth was 65% in eastern and southern Africa and reached 75% in western and central Africa [11].

Some women resume ovulation and menstruation as early as 28 days after birth [12]. Women who desire to prevent or delay a subsequent pregnancy after childbirth should commence a contraceptive method early after birth and before the resumption of sexual activity [12, 13]. However, gaps exist in meeting the demand for contraception among women of reproductive age, particularly in the early post-partum period [14].

The Ethiopian Minister of Health (MOH) is putting great efforts into programmatic and policy initiatives to increase access to and utilization of early postpartum contraceptive methods through the rapid expansion of primary health care facilities, massive training of health care workers, and free provision of family planning services [15]. Despite the national efforts, Ethiopia continues to have low uptake and high unmet need of early postpartum contraceptives; and a high rate of maternal and neonatal morbidity and mortality associated with abortion, pregnancy-related obstetric complications, childbirth, and postpartum complications [16]. Early postpartum contraceptive prevalence is still relatively low (19.1%) in Ethiopia and the total fertility rate (4.1 children per woman) is still high compared with 2.5 globally [17]. Early postpartum contraceptive methods are available free of charge in many public health facilities, yet many women do not use them due to social, cultural, or healthcare services constraints in Ethiopia, although research on these constraints is limited [18, 19].

In Ethiopia, though many quantitative studies have been conducted on postpartum family planning, the underlying individual, societal, and cultural barriers/challenges that hinder the uptake of early postpartum family planning methods have not yet been sufficiently explored. We aimed to explore the barriers/challenges that hamper the uptake of early postpartum modern contraceptive methods in northeastern Ethiopia. A better understanding of barriers/challenges affecting the adoption of early postpartum contraceptive methods is fundamental to the reproductive health programs, local planners, Policymakers, Non-Governmental Organizations (NGOs), and service providers to overcome the barriers and to come up with locally feasible and acceptable interventions. The study also provides baseline information for further study on the subject matter.

## Methods and materials

### Study context, period, and design

This study was conducted in the Dessie and Kombolcha town zones in Amhara regional state, Northeast Ethiopia. Dessie is also the administrative town of the south Wollo zone, which is situated 401 KM from Addis Ababa to the north. Dessie city is split into 5 sub cities with 22 kebeles which has 2 governmental hospitals and ten public health centers. Based on population projection for 2023 more than 700,000 resident population with an estimated 21,620 pregnant women. Kombolcha town is 30 km from Dessie city and 375 km from Addis Ababa that is an industrial zone and dry port in northeast Ethiopia. There are more than 350,000 resident populations with 16,100 estimated pregnant women. It is divided into 5 sub cities with 19 kebeles which have one governmental hospital and five health centers [20]. The study was conducted from December 15, 2022, to January 30, 2023. The qualitative research method specifically the conventional content analysis approach was applied.

### Participants and eligibility

To explore more about barriers and challenges to early post-partum contraceptive uptake, participants were purposively selected as information-rich sources. These included spouses or partners, health care providers, religious leaders, and postpartum women during 45–60 days after childbirth. Participants who lived six months or more in the area (permanent residents) were included in the study.

### Sample size and participant recruitment procedures

The sample size for the qualitative study was determined by reaching information saturation. The study involved 7 key informant interviews, 6 in-depth interviews, and 5 Focus Group Discussions (FDGs) with, a total of 13 interviewees and 44 discussants. To ensure a diverse representation, a mixed-purposeful sampling approach based on theoretical sampling was employed. This method involved selecting participants from the community with a combination of diverse characteristics such as age, educational status, residence, and occupation.

The selection criteria also considered individuals with a rich source of information and experience related to the barriers and challenges associated with the uptake of early postpartum modern contraceptives. Both the community and health facility in the study area were tapped into for participant selection. During data collection, those respondents perceived to have extensive insights into the study’s topic were prioritized for Key informants, in-depth interviews, and focus group discussions.

### Data collection tools, procedures, and techniques

Unstructured in-depth interviews and a Focus Group Discussion (FGD) guide were developed through an intensive review of documents related to barriers/facilitators of early postpartum family planning [18, 30, 39]. The guide was prepared in English and translated to the local Amharic language. The guide addressed diverse topics related to barriers/challenges of early postpartum family planning at the individual, community, cultural, religious, gender, and facility levels. Each of these major themes was accompanied by several possible probes and follow-up questions. Early communication with local health extension workers and pre-arrangement with key informants and focus group discussion participants was done.

When the participant agreed after informed consent to participate in the study, a meeting was scheduled at a time that was convenient for the participant. The location selected ensured the participant’s privacy and was mutually agreed upon. The interviewer initiated a "warm-up" ice-breaking discussion to reduce the tension among the FGD participants and requested permission to start the interview and audio recording to ensure verbatim transcription. The probing technique was applied to get further clarity from the participants and discussants and to get adequate data on barriers to the uptake of early postpartum modern contraceptive methods.

Anonymity was kept to minimize social desirability bias and no recording of their name. One moderator and one scribe participated per discussion and interview. Key informat interviews, In-depth interviews and Focus Group Discussions (FGDs) were conducted for a minimum of 45 and a maximum of 60 minutes. Data saturation was ensured when all questions were answered, responses were completed, and no further new information was obtained from various respondents. Redundancy was tolerated by up to three respondents. All data collection process was audio recorded by digital recorder besides note taking. A scribe was taking notes during the interview regarding body language, and verbal, and nonverbal cues and labeled the audio tape with the pseudonym. After all questions were addressed, the participants asked if there was anything additional they wanted to discuss or mention. The interviewer thanked them after the completion of the interview.

### Rigor and trustworthiness

The study trustworthiness was assured by based on Lincoln and Guba’s criteria of credibility, dependability, conformability, reflectivity, bracketing and transferability [21]. Credibility was assured by maintaining the consistency of procedures and the neutrality of the investigator about findings. Moreover, debriefing sessions with data collectors from different experiences were used to check the consistency. In addition to this the interview guide, transcription, coding process, and findings were shown to the qualitative research expert to cross-check and get feedback. During each interview, the data collectors observed participants’ body movements; facial expressions, eye gaze, and tone of speech each and everything was recorded by note takers.

To allow judgments about transferability by the reader; there was a rich description that can help the reader to understand the circumstances. During the interview, appropriate probing techniques were used. After data collection, the investigators transcribed the audio-recorded data in the participant’s local language in written form and then back-translated to English, and then the data were coded, categorized, and analyzed for ease of interpretation. To check conformability; raw data was tape-recorded during interviews. In addition, it was achieved by using quotes, which means, linking the words of the participants and with the discoveries. Throughout the study reflexivity and bracketing was assured by promoting self-awareness, critical self-examination, and transparency in the research process. By acknowledging and addressing the researcher’s role in shaping the study, reflectivity helps to ensure that the findings accurately represent the perspectives and experiences of the participants.

### Data management and analysis

The data were analyzed using conventional content analysis approach. The audio-recorded interview was transcribed verbatim immediately the same day, and translated into English language. The translated data were read line-by-line to understand the context and the meanings. Transcribed data were exported to ATLAS. Ti 7.1 software to produce meaningful codes, main categories and sub-themes. Sub-themes were reviewed further to develop overarching themes. Then the result findings reported with some important direct quotes. Interpretations of the qualitative data were dependent upon participants’ descriptions of their experiences and perceptions, which the researchers checked against the verbatim transcripts for accuracy and consistency.

### Operational definitions

#### Early postpartum modern contraceptive method uptake

Early postpartum modern contraceptive method use is outlined as women who have ever used any kind of modern birth control technique at intervals during the first six weeks after she gave birth [19].

#### Barriers/Challenges

Those underlying individual, cultural, social, and health facility factors that hinder the uptake of early postpartum modern family planning service uptake.

#### Culture/Traditions

Beliefs, customs, practices, and social behavior embedded in the community.

### Ethics approval and consent to participate

Ethical approval was taken from the Ethical Review Board (IRB) of Jimma University, the Institute of Health and Wollo University, department of Public health. A letter of permission was obtained from local Administrative offices. A written informed consent was obtained from participants before data collection. They were informed that participating in the study is voluntary. The right to withdraw from the study at any time was assured. Interviews were conducted in privacy and no personal identifier was recorded on the main questionnaire to protect the confidentiality and anonymity of study participants. Study participants were not identified by name in any report or publication from this study. All interviewees were informed about the objectives, and data collection procedures including using an audio recorder, possible risks and benefits of taking part in the research, and confidentiality of the obtained information. The audio interview and transcribed data were stored on a password-protected personal computer.

## Result

### Socio-demographic characteristics of participants

The study involved 7 key informants’ interviews, 6 in-depth interviews, and 5 Focus Group Discussions and a total of 13 interviewees and 44 discussants were participated. In-depth interview participants were health care providers, husbands, and religious leaders/teachers, whereas focus group discussion participants were mothers in the postpartum period. The participants’ ages ranged from 22 to 45 years. The majority of the participants 40.4% were 25–29 years old. Most of the participants 43.9% had primary education, 54.4% were housewives, 59.7% were Muslim and 98.2% were currently married. Among the study participants, 77.2% were postpartum women [**Table 1**].

**Table 1:** Socio-demographic characteristics of study participants on exploring barriers/challenges to uptake early postpartum contraceptive methods in northeast Ethiopia.

Characteristics	Category	Frequency	Percent (%)
Age	20–24 years	16	28.1
	25–29 years	23	40.4
	30–34 years	9	15.8
	35–45 years	9	15.8
Parity			
	1–3	49	86.0
	> = 4	8	14.1
Family size	1–4	40	70.2
	> = 5	17	29.8
Residence	Dessie	34	59.6
	Kombolcha	23	40.4
Sex of participant	Male	8	14.0
	Female	49	86.0
Religion of participant	Muslim	34	59.7
	Christian	23	40.3
Marital status of the participant	Single	1	1.8
	Married	56	98.2
Occupation of participant	Housewife	31	54.4
	Government employ	11	19.3
	Merchant	15	26.4
Participant category	Postpartum women	43	75.4
	Health worker	6	10.5
	Husband	4	7.0
	Religious leader	4	7.0
The educational level of the participant	No formal education	4	7.0
	Primary education	25	43.9
	Secondary education	18	31.6
	Higher education	10	17.5

### Themes of barriers to uptake of early postpartum contraceptive methods

The results were organized based on eight primary themes that highlight obstacles to adopting modern contraceptive methods in the early postpartum period. Participants in the study identified barriers to the acceptance of early postpartum contraception, which can be classified into eight themes: impediments related to knowledge, attitudes/beliefs, and family and community dynamics, health facility challenges, contraceptive methods related barriers, cultural hindrances, gender-related issues and misconceptions.

### Knowledge related barriers

The majority of interviewees indicated their awareness of modern contraceptive choices. However, a notable risk associated with unintended pregnancies and short intervals between childbirths is the insufficient awareness of early postpartum contraception. When specifically asked about early postpartum contraception, only half of the participants were familiar with it. Common barriers related to knowledge included a lack of understanding regarding the timing of pregnancy after childbirth, uncertainty about whether monthly bleeding or menstruation can signal pregnancy, and gaps in information regarding the possibility of becoming pregnant while breastfeeding. Mothers believe that pregnancy cannot occur if they do not experience monthly bleeding or menstruation following childbirth. The examples of FGD discussion excerpts from the conversations *“Unwanted pregnancies and short birth intervals are caused by a lack of knowledge about when pregnancy can occur following childbirth*. *Menstruation can sometimes vanish in a single area*, *leaving the woman unaware of it and believing that even after sexual initiation*, *she has not become pregnant*. *Women also think that I am breastfeeding and have no risk of pregnancy” [PPW*, *Dessie*, *FDG*, *24 years]*.

Commonly cited knowledge gaps related to contraceptive methods among breastfeeding mothers include a lack of information about options suitable for breastfeeding, limited awareness of methods available within the first 42 days after childbirth, insufficient understanding of the significance of early contraception, and a general lack of awareness about modern contraceptive options in the early postpartum period. The examples of excerpts from the KII conversations “*Knowledge gaps include things like not knowing that a contraceptive method can be taken 42 days after giving birth and clients are unaware that there are methods available that are pleasant for breastfeeding mothers*. *There may be information gaps that influence the early postpartum uptake of modern contraceptive methods due to low acceptance or disregard for counseling for early contraceptive method uptake during prenatal care" [KII*, *HW*, *Kombolcha*, *35 years]*.

### Attitude/Belief barriers

The prevailing attitude-related barriers to the early adoption of modern postpartum contraception include beliefs that modern contraceptives can dry up breast milk, the perception that breastfeeding prevents pregnancy, a strong inclination to avoid a narrow birth interval after a cesarean section delivery, and the perceived low likelihood of fertility after childbirth. Examples of excerpts from the discussions, "*There is a belief that women won’t become pregnant if she breastfeeds and that using modern contraceptives weakens the hands and makes it harder to perform strenuous tasks*. *The participant said*, *"It seems to me that some women also think that contemporary forms of contraception dry out breast milk*, *which is why they don’t take them early" [FGD*, *PPW*, *Dessie*, *25 years]*.

### Family/Community barriers

As per the feedback from participants, obstacles related to family that hinder the adoption of early postpartum modern contraception include a lack of family support, forgetting or failing to remember to take the contraceptive method promptly, husbands expressing reluctance towards contraceptive use, and the perception that preventing a narrow birth interval is primarily the responsibility of the woman. Instances of excerpts from the conversations, “*If the woman has a workload at home after childbirth she may not remember/ forget to take an early postpartum contraceptive method*. *I faced challenges in taking the method from a health facility due to lack of family support*, *for example*, *I delivered twins and faced difficulty in caring for children when I wanted to go to a health facility to take the method*. *My husband’s mother was not cooperative in supporting and caring for the children*, *so I requested another person to help me and take the method to prevent unwanted pregnancy” [FGD*, *PPW*, *Dessie*, *30 years]*.

### Health facility barriers

#### Providers approach and poor counseling

The responses from participants highlight prevalent obstacles within health facilities to the early adoption of modern postpartum contraception. These include insufficient counseling during antenatal, delivery, and postnatal care regarding early postpartum modern contraception. Another identified barrier within health facilities pertains to the approach and communication of healthcare providers. In the course of the in-depth interview (IDI), the participant expressed that, **the "***health care provider’s capacity and approach to deliver services was disappointing*. *He said also health providers did not tell us when and how she would take birth control methods after childbirth*. *They do not have the skill to provide the service*, *and they do not have drug choices [IDI*, *husband*, *Dessie*, *35 years]*. A different participant in the Key Informant Interview (KII) mentioned that “*health facility barriers*, *some trained health professionals not delivering the service due to skill gaps*, *Poor counseling during antenatal care about early postpartum modern contraception*, *and providers’ approach during antenatal care and delivery can be health facility gap to promote mother about the uptake of early postpartum modern contraceptive method” [KII*, *HW*, *Kombolcha*, *30 years]*.

#### Lack of reminder and follow-up mechanism

A frequently mentioned hindrance to the early adoption of postpartum modern family planning methods is the absence of reminders and follow-up mechanisms during and after childbirth. This challenge is emphasized as a barrier to preventing unwanted and narrow birth intervals. Instances of excerpts from the conversations, **“***Previously there was continuous information*, *education and communication on family planning*, *but in last 3–4 years there is no education on contraceptive methods and benefits*, *this may make mothers not take early postpartum contraceptive methods early to prevent unwanted pregnancy"[FGD*, *PPW*, *Dessie*, *29 years]*.

#### Method choice and availability barriers

Participants identified ignorance regarding contraceptive method options provided by healthcare providers, as well as a scarcity of available choices and limited method availability at health facilities, as barriers to the early adoption of postpartum modern contraceptive methods. Participants in the Focus Group Discussion (FGD) mentioned that "*health care providers do not give us method choice of contraceptive methods*. *Example Before I gave birth*, *I asked them to give me the contraceptive method of injection that can be taken every 3 months*, *but they said the method was expired and they have no injection*. *Then they gave me implants under my arm which is without my choice*. *After this I did not take any contraceptive method" [FGD*, *PPW*, *Kombolcha*, *22 years]*. Another participant in the in-depth Key Informant Interview (KII) mentioned that,*” all methods may not be stocked out from the health facility*, *but sometimes choices may not be available due to shortage of methods” [KII*, *HW*, *Dessie*, *40 years]*.

#### Health facility administrative service accommodation barriers

According to the study participants, administrative obstacles to the early adoption of postpartum modern family planning methods from health facilities were commonly grouped into subthemes. These included issues related to prepayment and card draw procedures, extended waiting times at health facilities, the considerable distance to health facilities, barriers associated with service delivery and opening hours, and errors made by health workers. Participants in the in-depth interview (IDI) mentioned that “*administrative accommodation barriers like client card draw procedure*, *long waiting times for the service at a health facility*, *service delivery and opening time not being convenient and service delivery room problems are the common service administrative barriers at health facilities to uptake early postpartum contraceptive methods after childbirth” [IDI*, *husband*, *Dessie*, *40 years]*.

#### Method related factors

Participants were inquired about obstacles related to the adoption of family planning methods shortly after childbirth. Frequently cited barriers in this regard include health concerns regarding potential side effects, anxiety about method failure, fear of adverse effects, rumors suggesting that contraceptive methods could lead to infertility, and the challenge of remembering to take daily method pills. A participant in the Key Informant Interview (KII) mentioned that “*clients raised many complaints about the method’s side effects like implants and IUCD can disappear from the body*, *weakness in the hand to do the job*, *hair loss*, *change face color/madat and fertility delay/ infertility*, *especially injectable/depo*. *The participant also explained that the mother said*, *I gave birth last year and am not capable of my health to take the method until the child grows some*. *" Mothers also said I need rest from hormonal methods*, *side effects like face color change/madat*, *irregular bleeding or hand weakness” [KII*, *HW*, *Dessie*, *40 years]*.

#### Cultural and religious barriers

Participants were queried about cultural impediments to the adoption of early postpartum contraceptives. The identified themes included the perception that early uptake implies early sexual initiation, leading to social stigma and fear, which is culturally sensitive and labels the woman with highly sexual feelings. Additionally, cultural home practices that keep the woman occupied, the cultural unacceptability of leaving home 40 days after childbirth, leading to potential health issues, a cultural promotion of having more children, and religious restrictions and perceptions towards contraception were recognized as cultural and religious barriers to the early adoption of postpartum contraception. FGD participant said, “*If the woman takes contraceptive methods during the early postpartum period*, *other people perceive that she starts sexual intercourse immediately after childbirth which leads to fear of social desirability bias*. *Due to fear of this*, *they may start sexual intercourse hiddenly without contraception which leads to unwanted pregnancy and narrow birth interval*. *The participant also mentioned that going out of the home 40 days after childbirth is culturally not acceptable in the community due to fear of evil"[FGD*, *PPW*, *Dessie*, *26 years]*.

Another participant in the in-depth interview mentioned, “*I am Muslim*, *and birth control methods are not accepted in my opinion and religious thought*. *If the woman has no health problem no need to use contraceptive methods*. *The participant explained that; because the use of birth control methods is considered as killing the life of human beings in Sharia thought*. *If the pregnancy affects the health of the woman; it is possible to use contraceptives and extend 3–4 years the next pregnancy” [IDI*, *religious teacher*, *Dessie*, *35 years]*.

#### Gender-related barriers

The participants in the study identified gender-related obstacles to the early adoption of postpartum contraception. These include husbands expressing a frequent desire for childbirth and lack of support for contraceptives, decision-making primarily being the responsibility of males and the fear of husbands, low male involvement and responsibility in family planning, husbands showing disinterest in discussing family planning, male opposition and conflicts regarding the uptake of early postpartum modern contraceptives, preferences for the sex of the child, and the perception by males that the responsibility for adopting early postpartum contraception and preventing a narrow birth interval lies with the woman. Examples of excerpts from the discussions “*males are not responsible and participatory to family planning*. *For example*, *the recent neighbor was delivered a year ago*, *now she has given birth again after a year*. *Due to this*, *her husband blamed her*, *why you get pregnant with this short birth interval*. *Then they divorced due to this unwanted pregnancy and she now cares for the babies with the small payment of washing clothes of other people" [FGD*, *PPW*, *Dessie*, *36 years]"*. Another KII participant said “*Women are dependent on their husbands and sex preference is a challenge to uptake modern contraceptive methods early after birth*. *For example*, *from my experience*, *a 35-year-old had 9 children and came from our catchment area for delivery*. *I asked her why you have this number of children at this age*. *She said to me all are female sex and need of the male child to satisfy her husband” [KII*, *HW*, *Kombolcha*, *38 years]*.

#### Misconceptions barriers

Participants were questioned about misconceptions surrounding the adoption of early postpartum modern family planning and frequently mentioned barriers included misunderstandings such as the belief that modern contraceptive methods can dry up breast milk, the concern that modern contraceptives may lead to infertility, misconceptions about intrauterine contraceptives devices (IUCD) disappearing or protruding from the body and causing unwanted pregnancies, misunderstandings related to method side effects, and misconceptions about contraceptive failure. Examples of excerpts from the discussions, “*Second-hand reports like contraceptive methods inserted under the arm can cause difficulty to do the job and affect health*, *makes the body thin and causes bleeding are the common rumors that can be a barrier to uptake modern early postpartum modern contraceptive methods” [PPW*, *FGD*, *Kombolcha*, *35 years]*.

#### Conceptual model and network analysis of barriers to uptake of early postpartum contraceptive method

**Fig 1:**
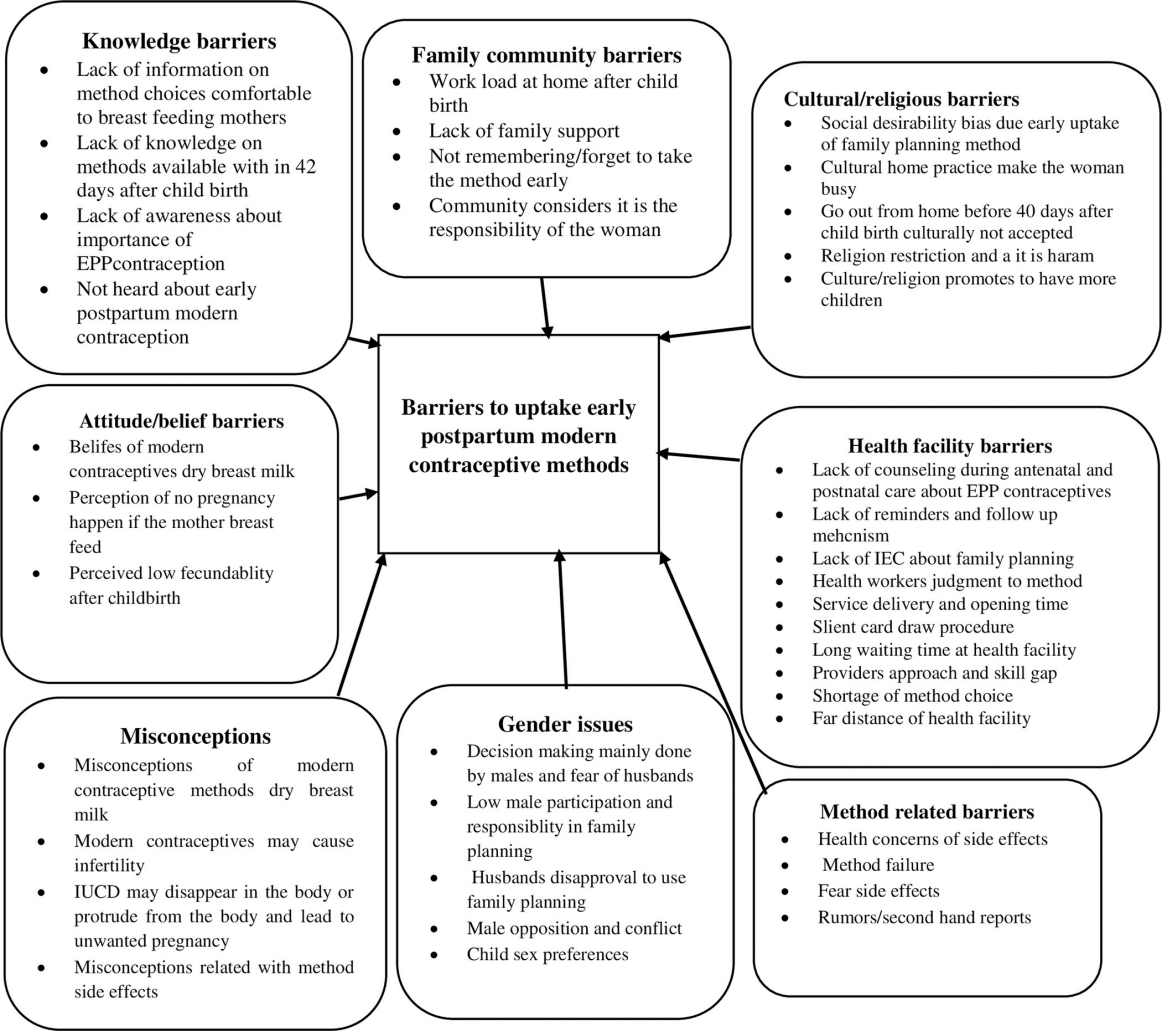
Conceptual model and network analysis of barriers to the study exploring barriers and facilitators of early adoption of postpartum modern contraceptive methods after childbirth in northeast Ethiopia.

Participants in the study identified barriers to the adoption of early postpartum modern contraceptive methods, which can be grouped into various overarching themes. These include barriers related to knowledge, attitudes/beliefs, family and community dynamics, health facilities, method-related factors, cultural aspects, gender issues, and misconceptions [**Fig 1**].

#### Participant’s suggested facilitators of early postpartum modern contraceptive method uptake

Participants proposed strategies to enhance the uptake of early postpartum modern contraceptives. These strategies involve implementing information, education, and communication initiatives to raise awareness about the likely time of conception after childbirth and the availability of safe modern contraceptive methods suitable for breastfeeding mothers. In addition, participants recommended addressing health facility barriers through measures that streamline the bureaucracy for family planning service access, promote male participation and personal responsibility for early postpartum modern contraceptive methods, and develop modern contraceptive methods and technologies specifically designed for males to prevent narrow birth intervals, thereby playing a critical role in reducing preventable maternal and newborn morbidity and mortality [**Table 2**].

**Table 2:** Summary of suggestions for improving uptake of early postpartum family planning in northeast Ethiopia.

Barriers	Solutions/facilitators
Knowledge and attitude barriers	• IEC on time of pregnancy happening after childbirth • Health Education on the availability of modern contraceptive methods of choice that are safe for breastfeeding mothers. • Couple counseling and education about birth spacing and early postpartum contraception during pregnancy follow-up, childbirth, and after childbirth; • Early reminders and follow-up mechanisms by birth facilities that are locally feasible and acceptable after childbirth about early postpartum contraception and birth spacing. • Promotion of birth spacing and early postpartum contraception through different media like radio, television, social media, and printed materials • Coaching and mentorship to trained healthcare providers
Family community barriers	• Family/community discussion on early postpartum contraception and birth spacing • Community forums to social support of postpartum women at home • Educate them about the dangers of the narrow birth interval in the community
Health facility barriers	• Avoid health facility bureaucracy client card draw procedure, and convenient service opening time to clients. • Family planning service integration with antenatal, delivery, postpartum care, and child immunization services • Training of health care providers on client center approach and counseling • Avoid long waiting times at health facilities to uptake the service • Build a strong family planning commodity security system
Cultural and religious barriers	• Awareness creation to religious leaders/teachers, husbands, and the public about birth spacing and contraception and, the dangers of narrow birth intervals. • Health workers give talks in churches, on mass media
Method related barriers	• Improve management of side-effects • Develop and innovate minimal side effects and long-lasting contraceptive methods both for women and men options.
Gender issues	• Improve male responsibility and participation • Improve women’s decision-making power on their health, • Awareness of gender equality and avoiding girl-child discrimination.
Misconceptions	• Expert users to raise awareness and address concerns • Counseling and education to avoid rumors and second-hand reports

An In Depth Interview Participant mentioned that *“promotion through different media*, *information*, *education and communication to the religious leaders*, *husbands*, *and community about early postpartum family planning and birth spacing*, *follow up and reminders after childbirth and fulfill the service at the nearby health facility can enhance uptake of early postpartum modern contraceptive method” [KII*, *husband*, *Kombolcha*, *35 years]*. Another excerpts from the discussions,”*to improve early postpartum contraceptive uptake the health facility should avoid bureaucratic client card draw procedure*, *need of family planning service integration at different service unites like antenatal care and immunization*, *avoid long waiting time to the service*, *strong education and counseling during antenatal care about early postpartum contraception and reminder system and follow up mechanisms after child birth about uptake of early postpartum contraceptive methods to women’’[KII*, *PPW*, *Dessie*, *40 years]*.

## Discussion

Findings from this study reveal the influence of cultural and religious factors on contraceptive uptake as expressed by both participants and healthcare providers in the study area. This conforms to other literature in other parts of Sub-Saharan Africa [22, 23]. Social fear and religious restrictions that do not allow women to go out of their homes to access early postpartum contraceptive methods require social behavioral change interventions linked with family planning programs and strategies. This study also pulled out gender-related barriers to early postpartum contraception which included husbands’ need for frequent births and therefore do not support contraceptives with decision-making mainly done by males. The fear of husbands, low male participation and responsibility, male opposition and conflict to uptake early postpartum modern contraceptives, and child sex preferences were the main barriers to female contraceptive uptake. This study is consistent with known findings from other studies from other parts of Africa [24–26]. Fostering a common understanding of the dangers of a narrow or short birth interval in the community and the need for appropriate interventions to educate men about the reproductive health needs of their wives is key.

In this study, participants reported that trained staff shortages, providers’ approach and interaction, and service provider’s communication and commitment are major challenges at the health facilities and this is attributed to long waiting times at the health facilities. In many of the facilities, only one provider was available to provide family planning and other services, a situation that often led to providers being overworked under pressure and prone to exhaustion. Trained manpower shortages in the right number and skill mix have been documented to be a major obstacle in the adoption of early postpartum family planning services in northeast Ethiopia. This finding is similar to other literature on similar settings in sub-Saharan Africa [27–29]. Efforts have been made by Ethiopia’s minister of health to expand primary healthcare facilities, but service quality, provider motivation, and capacity-building programs still need attention to improve early postpartum modern family planning programs.

Women had myths and misconceptions about early postpartum family planning methods. For instance, modern contraceptive methods are said to dry breast milk and may cause infertility, IUCD may disappear in the body or protrude from the body, and perception that IUCD is not comfortable for sexual intercourse were barriers to early uptake of this postpartum family planning method. The finding is supported by previous studies [29–31]. Such misconceptions should be addressed by behavioral interventions, mass media communication, and community engagement communication strategies that deal with myths and misconceptions. This solidifies the need to comprehensively sensitize family planning users about possible side effects and thereby address FP myths and misconceptions that exist. The study also explored that beliefs like; modern contraceptives dry breast milk, perceived low fecundablity after childbirth, and low acceptance of counseling during pregnancy about early postpartum modern contraception are attitude barriers to early uptake of postpartum family planning. This goes in line with the findings from other studies which revealed that fears, misconceptions or misinformation, and side effects (actual or perceived) of methods were common barriers to the adoption and continuation of modern contraception [29, 32, 33]. In addition, the knowledge gap on early postpartum family planning leads to the propagation of myths and misconceptions among women.

Findings from this study showed that lack of early postpartum contraception-specific knowledge emerged as an important barrier to the early adoption of postpartum modern family planning. The most commonly identified knowledge gap-related barriers were if the mother breastfeeds pregnancy can not occur if menstruation is not seen regularly and pregnancy cannot occur, lack of knowledge on time to take birth control methods after childbirth, not knowing the time of pregnancy happening after childbirth, lack of information on method choices available to breastfeeding mothers and lack of knowledge on methods available within 42 days after childbirth, most women believe they are not at risk of becoming pregnant and need time to recover during early postpartum period. Other previous literature also supports this finding [28, 34]. This indicates the need for educational interventions through facility-based, community-based, and different Media can help increase knowledge of available methods, enabling individuals to make informed choices and use FP more effectively.

The findings of this study reveal health facility barriers to early uptake of family planning methods explored by both clients and providers were lack of counseling during antenatal, delivery, and postnatal care, lack of reminders and follow-up mechanisms during and after childbirth about early postpartum contraceptives, health workers judgment on contraceptive method choice, shortage of methods availability at a health facility, health facility service delivery and opening time not convenient, client card draw procedure, health facility administrative accommodation barriers, long waiting time at a health facility, providers approach and skill gap, a far distance of health facility and mistakes from health workers. This conforms with other study findings which hinder early uptake of postpartum family planning [35–37]. Based on our study findings we suggest that greater consideration be given to focusing on antenatal, childbirth, and early postpartum contraceptive counseling. Such counseling may be an effective strategy to increase the use of birth-spacing contraceptives. Health facilities also need to improve service delivery bureaucratic barriers, method choice availability, and capacity building to service providers to resolve health facility barriers that hinder early postpartum modern contraceptive method use.

This study’s findings reveal health concerns of actual method side effects like persistent menstrual bleeding which is problematic when women cannot afford to buy extra sanitary products. Additionally, when their husbands view this as a barrier to a sexual relationship, change of face color (Madat), thinness of body/weakness (implants), no return of fertility after method discontinuation (Depo Provera), method failure and perceived fear of method side effects were reported method related barriers to the use of early post-partum modern contraceptive methods. Studies from different regions across the globe also show similar findings, indicating that these barriers are global challenges to the early uptake of family planning methods[38–40]. This highlights the importance of addressing concerns through side effects management, reassurance, and innovation of new minimal side effects, easy-to-use, and long-lasting contraceptive methods to increase uptake.

### Strength and limitations of the study

This qualitative study provided a broader exploration and deeper understanding of the findings to barriers/facilitators; we elicited information by self-reporting from respondents who were prone to selection bias due to purposeful selection of participants that may lack representation and social desirability bias due to the sensitive nature of the issue in the community.

## Conclusion

The type of barrier a woman faces in the adoption of early postpartum modern family planning services is a product of not only her characteristics but is influenced by the characteristics of her household, health facilities service delivery and accommodation process, providers’ approach, lack of reminder and follow up mechanisms after childbirth, cultural and religious objections, gender-related issues, method related rumors, side effects, second-hand reports; and misconceptions. The availability of family planning commodities was not consistent at health facilities. Lack of male willingness, participation, and responsibility was also a big challenge to the adoption of early postpartum contraceptive methods.

### Recommendations

Policymakers should design interventions that address barriers of reminders and follow-up mechanisms such as mobile health interventions, social networking, fast health facility service accommodation systems and meaningfully engagement of male partners.

Regional and zonal health bureaus should develop pragmatic and integrated strategies to improve early adoption of postpartum contraceptive methods at health facilities, this should be a priority area of action. Improve contraceptive method safety and training of health care providers on client-centered care and counseling about early postpartum family planning.

Health facility family planning service providers work on information and education on fertility return time after childbirth, benefits of initiation of contraception before the return of menses or resumption of sexual activity, provide a method of choice and client-centered care, strengthening family planning counseling, integrating early postpartum family planning with maternal and child health service delivery. Health facilities should improve bureaucratic client card draw procedure, service delivery and opening time; and follow-up mechanisms after childbirth.

Local community health workers should focus on couple counseling on family planning, massive early postpartum contraception awareness to women and their social support systems to avoid myths and misconceptions, Community-based systematic and culturally sensitive peer education programs should be implemented to improve the perceptions, increase risk awareness of women and families about narrow birth interval complications, and improve restrictive cultural and religious practices.

Researchers should focus on innovation and development of minimal side effects, safe and long-lasting contraceptive methods both for women and men options to resolve method side effect barriers of available methods and resolve gender barriers around women-centered contraception.

## Supporting information

S1 Dataset Data sets to the study barriers and facilitators of early postpartum modern contraceptive method uptake in Dessie and Kombolcha City zones, northeast Ethiopia. (ZIP)

## List of acronyms/abbreviations

ANC: Antenatal Care
EDHS: Ethiopia Demographic and Health Survey
EPPMFP: Early Post-Partum Modern Family Planning
FGD: Focus Group Discussion
IEC: Information Education and Counseling
IUCD: IntraUterine Contraceptive Device
KII: Key Informant Interview
HW: Health Worker
PPW: Postpartum Women
MOH: Ministry of Health
FP: Family Planning
NGO: Non Governmental Organizations
HEP: Health Extension Program
PI: Principal Investigator
SDG: Sustainable Development Goals
TBA: Traditional Birth Attendants
WHO: World Health Organization
