# Supplementary material for: Barriers and facilitators of early postpartum modern contraceptive method uptake in Dessie and Kombolcha City zones, northeast Ethiopia: Conventional content analysis qualitative study
Source: PLoS One. 2024 Jul 17;19(7):e0305971. doi: 10.1371/journal.pone.0305971 (PMC11253950; doi:10.1371/journal.pone.0305971)
Supplement: S1 Dataset — (ZIP) [file pone.0305971.s001.zip › Supporting information file/IDI_KII and FGD Transcriptions/FGD_Transcription_BW_1_Niguss Cherie.docx]

**Exploring barriers/challenges to early postpartum contraceptive method uptake among women**

Region: **Amhara**

Zone/Town: Dessie

Cluster/Sub city: Buanbawuha

Location: **North Ethiopia**

Participant category:

Interviewer name: Niguss Cherie

Transcriber name: Niguss Cherie

Date: 22/11/2022

Start time: 4:30

End time: 5:35

Duration: 65

**FGD transcriptions of conversions –Buanbawuha_NC_1**

| Discussant code | Age | HH condition | Religion | Educational background | Parity | Occupation | Residence | Family size | Zone |
| --- | --- | --- | --- | --- | --- | --- | --- | --- | --- |
| P1 | 22 | R | C | College | 1 | Nurse | K10 | 3 | Dessie |
| P2 | 24 | O | C | 2ndary | 2 | HWife | K10 | 4 | Dessie |
| P3 | 26 | O | M | Primary | 1 | HWife | K10 | 3 | Dessie |
| P4 | 25 | R | M | 2ndary | 3 | Merchant | K10 | 4 | Dessie |
| P5 | 28 | O | C | 2ndary | 2 | HWife | K10 | 5 | Dessie |
| P6 | 23 | R | M | College | 3 | Merchant | K10 | 4 | Dessie |
| P7 | 25 | O | M | Primary | 2 | HWife | K10 | 4 | Dessie |
| P8 | 29 | R | C | 2ndary | 3 | Gov.t W | K10 | 5 | Dessie |

**R= Rent O: Own C:Chrstian M: Muslim**

I: Do you heard about early postpartum family planning?

P1: We heard, but we do not know it.

P3: I do not know it.

P5: I did not hear about it.

I: When a woman can be pregnant after child birth?

P1: After showing monthly bleeding next to child birth.

P7: The woman can get in pregnant after a month of child birth.

I: What is the ideal time to get pregnant to a woman after child birth?

P1, 2, 3: After 3 years of child birth

P 5, 6, 8: After 2 years of child birth

I: How do you comment birth spacing in your communiy?

P3: Now day’s people have information about family planning methods. But, sometimes I saw women give birth with short interval.

P5: Today there is challenge in economy to live and educate children. Everybody knows this, but sometimes unwanted pregnancy happens due to different reasons.

I: What is your role in early postpartum family planning? (**Probe :**)

**I: Do you discuss family planning with your partner/ spouse?**

P1, 5, 6: These participants said that, yes, they discuss about family planning with their husband.

P2, 3, 4,7, 8: They reported husbands are not interested to discuss on this issue.

**I: What are your views concerning family planning in general?**

P2: They are important to balance our economy with family size

P5: They are important to the health of the child and the mother

P6: Their said effect makes discomfort like thinness, hair loss and bleeding.

I: How do you feel about your partner/ spouse using family planning?

P6: Males need to birth frequently and not support contraceptive

P8: He is cooperative to use contraceptive methods.

**I: How comfortable are you to use family planning?**

P2: It is good to the health of the family

P4: I used before, but it makes me thin and when I change the method to pills it makes hair loss. Due to this I discontinued and get in unwanted pregnancy.

**I: Would you please mention facilitating factors (if any) to uptake early postpartum family planning?**

P5: Many people want to use the methods, but there is knowledge gap. This indicates need of strong counseling and education from health care workers.

P7: Women need to control their fertility, but some husbands are not cooperative and needs short birth intervals. Male participation and cooperativeness is important to facilitate the uptake of early postpartum modern contraceptive methods.

**I: Would you please explain challenges and barriers encountered to early postpartum family planning? Probe**

**I: Knowledge** (Probe: when pregnancy can happen? birth spacing? methods? where to get the service?)

P1: If the woman breast feed, mothers believe no pregnancy occurs within 6 months. This the main reason to not taking early postpartum modern contraceptive method.

P2: If I do not saw monthly bleeding/ menstruation after child birth, mothers think pregnancy not happen. When the woman waits her monthly bleeding, sometimes she gets in unwanted pregnancy.

P4: There is gap on information when to take birth control method after child birth.

P5: Not knowing the time when pregnancy can happen after child birth is the reason not taking early postpartum modern contraceptive method.

P7: Lack of information about the choices of methods which are comfortable to breast feeding mother.

P8: Women have also lacks knowledge on availability of the method that can be taken within 42 days after child birth at health facilities.

**I: Challenges related to family** (Probe: work load, family care,

P1: No

P2, 3, 4: No

**I: Attitude** (probe: opposing, method suitablity, Perceived low fecund ability)

P1: Yes, there is perception of get in pregnancy, if she feeds breast milk up to six months.

P2: Some women also belief that modern contraceptive methods dry breast milk, due to this they do not take contraceptive methods early.

**I: Health facility barriers** (service quality, administrative accommodation barriers, providers approach, choices, distance, counseling, IEC, privacy, interaction on family planning during pregnancy, child birth and after birth reminders...)

P1: No problem

P2: If the woman needs to take the method health workers deliver the method.

P3: Health workers educate about breast feed to protect unwanted pregnancy; this may delay to take early postpartum modern contraceptive methods.

P4: If health workers give strong counseling during antenatal care women can take early postpartum modern contraceptive methods.

P6: There is lack of reminders during and after child birth to the mother to take early postpartum modern contraceptive methods from health care facilities.

P8: **“Previously there were continues information, education and communication on family planning, but last 3-4 years there is no education on contraceptive methods and benefits, this may make mothers not taking early post-partum contraceptive methods early to prevent unwanted pregnancy”.**

**I: Method-related factors** (Health concern, accesses, and side effects)

P2: Yes, due to the method side effects mothers fear to take early post-partum modern contraceptive, For example I used post pill, but I get pregnant when the 1^st^ child age is 1 year.

P3: Health side effects of contraceptives can be another reason not to use early postpartum modern contraceptive methods. Example “**I used Injectable that was taken every 3 months on my arm, but that makes my body thin, then I shift to pills that were taken every day which affects loss of my hair. Then I stop the method and gets pregnant to give birth without willingness”**

P4:

**I: Cultural barriers** (Probe: encourage high number of children, Social desirablity fear, postpartum practice at home,)

P3: Cultural barriers can be barriers to uptake early postpartum modern contraceptive methods from health facilities. If the woman takes contraceptive methods during postpartum period, other people perceives that she starts sexual intercourse immediately after child birth which leads social desirability bias. This makes people the woman is sexy that is socially sensitive in our culture.

P4: Cultural home practices after child birth makes busy the woman and goes out of the home before 40 days after child birth is culturally not accept in the community. This can be a reason not to take contraceptive methods early after child birth.

**I: Gender issues** (Probe: Women’s empowerment, male engagement, husband opposition and contraceptive decision making)

P6: Husbands need to give birth in short interval and male participation to uptake early postpartum contraceptive methods is low.

P7: Contraceptive method uptake decision making mainly done by males and some husbands are opposing using contraceptive methods.

I: **Financial barriers** (probe: perceived expense of contraception,

P: This is not the problem.

**I: Fertility related factors** (Fertility Preferences, birth spacing, fertility intention...)

R: Child sex preference can be also a reason to not to uptake early postpartum contraceptive methods. “If the women give birth female sex or male sex child consecutively the couples are not interested to uptake early postpartum modern contraceptive methods. They have fertility intention without birth spacing to get the missed male of female sex child and do not use early postpartum contraception.

**I: Misconceptions** (probe: Rumors, secondhand reports of side effects?

P2: Misconceptions of conception can cause reduction of breast milk can be a barrier to uptake early postpartum contraceptive method uptake.

P3: Rumors related with contraceptive methods causes infertility is another reason not to uptake early postpartum modern contraceptive methods.

P6: Misconceptions related with IUCD like causes bleeding, disappear in to the body after insertion is a reason not to take early postpartum modern contraceptive methods.

P8: Second hand reports like contraceptive methods inserted under the arm can cause difficulty to do job and affects health, makes the body thin and causes bleeding are the common rumors that is barrier to uptake modern early postpartum modern contraceptive methods.

I: What do you suggest to enhance early postpartum family planning? How?

P1: Need of health education about fertility time after child birth and contraceptive method choices.

P3: Strong counseling during pregnancy related with early postpartum modern contraceptive methods can improve uptake early postpartum modern contraceptive methods.

P5: Early reminders and follow up after child birth also about early postpartum modern contraception.

P7: Continues information, education and communication about birth spacing, method choices and dangers of narrow birth interval.

I: Thank you! I have finished my questions. Do you have anything to add?

**R: No, Thank you.**

**I: Thank you very much!**

**End**

**Interviewer impression/comments**

The FDG was good in which the participant response looks open and honest. The participant involved with great interest and their participation level was cooperative. The discussion was completed without any interruption and no any disturbance or noisy happened.
