# Supplementary material for: Barriers and facilitators of early postpartum modern contraceptive method uptake in Dessie and Kombolcha City zones, northeast Ethiopia: Conventional content analysis qualitative study
Source: PLoS One. 2024 Jul 17;19(7):e0305971. doi: 10.1371/journal.pone.0305971 (PMC11253950; doi:10.1371/journal.pone.0305971)
Supplement: S1 Dataset — (ZIP) [file pone.0305971.s001.zip › Supporting information file/IDI_KII and FGD Transcriptions/FGD_Transcription_Kocha 05_5_Niguss Cherie.docx]

**Exploring barriers/challenges to early postpartum contraceptive method uptake among women**

Region: **Amhara**

Zone/Town: Dessie

Cluster/Sub city: Kocha 05

Location: **North Ethiopia**

Participant category: **Postpartum mothers**

Interviewer name: Niguss Cherie

Transcriber name: Niguss Cherie

Date: 22/11/2022

Start time: 10:30

End time: 11:30

Duration: 60 minutes

**FGD Transcriptions of conversions –Kocha 05_NC_5**

| Discussant code | Age | HH condition | Religion | Educational background | Parity | Occupation | Residence | Family size | Zone |
| --- | --- | --- | --- | --- | --- | --- | --- | --- | --- |
| P1 | 22 | R | M | 6 | 1 | HWife | K08 | 3 | Dessie |
| P2 | 25 | O | M | 8 | 1 | HWife | K08 | 3 | Dessie |
| P3 | 24 | R | M | 7 | 1 | HWife | K10 | 3 | Dessie |
| P4 | 25 | O | C | 5 | 2 | HWife | K10 | 3 | Dessie |
| P5 | 25 | R | M | 6 | 2 | HWife | K08 | 3 | Dessie |
| P6 | 26 | R | M | 7 | 2 | HWife | K10 |  | Dessie |
| P7 | 35 | O | M | 4 | 4 | HWife | K08 | 4 | Dessie |
| P8 | 22 | O | M | 8 | 2 | HWife | K08 | 3 | Dessie |
| P9 | 24 | R | M | 7 | 1 | HWife | K10 | 3 | Dessie |
| P10 | 25 | O | C | 5 | 2 | HWife | K10 | 3 | Dessie |

**R: Rent O: Own C: Christian M: Muslim**

**I: Do you heard about early postpartum family planning?**

P1, 2: The participants said, they did to hear about early postpartum modern contraceptive methods.

P3, 4, 5: They reported that, they heard about early postpartum modern contraceptive methods before.

I: When a woman can be pregnant after child birth?

P1, 3, 5, 8: The participants said, the woman can get pregnant after 45 days of child birth.

P2, 4, 7, 8: They reported, I f the woman breast feed she can get in pregnant after 2 years of child birth.

**I: What is the ideal time to get pregnant to a woman after child birth?**

P1, 2, 4, 5: They said, the ideal time of pregnancy after child birth is after 2 years of child birth.

P3, 6, 7: They said, it is good the woman gets pregnant after 3 years of child birth.

P8: She said the ideal time to the woman to get in pregnancy is after 4 years of child birth.

**I: How do you comment birth spacing in your community?**

P3: Now day’s people have information about family planning methods. But, sometimes I saw women give birth with short interval.

P5: Today there is challenge in economy to live and educate children. Everybody knows this, but sometimes unwanted pregnancy happens due to different reasons.

I: What is your role in early postpartum family planning? (**Probe :**)

**I: Do you discuss family planning with your partner/ spouse**?

P1: No

P2: No

P3: Yes

P4: No

P5: Yes

P6: Yes

P7: No

P8: Yes

**I: What are your views concerning family planning in general?**

P2: Their said effect makes discomfort like thinness, hair loss and bleeding.

P5: They are important to the health of the child and the mother

P6: They are important to balance our economy with family size

**I: How do you feel about your partner/ spouse using family planning?**

P6: He is cooperative to use contraceptive methods.

P8: Males need to birth frequently and not support contraceptive

**I: How comfortable are you to use family planning?**

P2: It is good to the health of the family

P4: I used before, but it makes me thin and when I change the method to pills it makes hair loss. Due to this I discontinued and get in unwanted pregnancy.

**I: Would you please mention facilitating factors (if any) to uptake early postpartum family planning?**

P5: Many people want to use the methods, but there is knowledge gap. This indicates need of strong counseling and education from health care workers.

P7: Women need to control their fertility, but some husbands are not cooperative and needs short birth intervals. Male participation and cooperativeness is important to facilitate the uptake of early postpartum modern contraceptive methods.

I: Would you please explain challenges and barriers encountered to early postpartum family planning? **Probe:**

**I: Knowledge** (Probe: when pregnancy can happen? birth spacing? methods? where to get the service?)

P5: The participant said, knowledge gap on the time of pregnancy happening after child birth many women gets in unwanted pregnancy.

P3: This participant reported that, women think that I am breast feeding and no risk pregnancy

P8: The participant said women said, if monthly bleeding/ menstruation not seen after child birth I am no probability of pregnancy. Due to this they did not take early postpartum modern contraceptive methods.

P7: This participant said even women take contraceptive methods some women gets pregnant after taking birth control methods. The contraception method has problem not preventing pregnancy. **For example “a woman at my neighbor takes contraceptive method under her arm, but she gets pregnant. After this I did not take any contraceptive method.”**

**I: Challenges related to family** (Probe: work load, Family support)

P1: This participant said, if the woman has work load at home after child birth she may not remember/ forget to take early postpartum contraceptive method. Due to this reason unwanted pregnancy can happen with short birth interval.

P3: The participant explained that, if there is comfort at home after child birth, the woman may need to pregnant again

**I: Attitude** (probe: opposing, method suitablity, Perceived low fecund ability)

P2: She explained that, if the woman delivers by delivery by C/S, health workers counsel strongly to fear narrow pregnancy that may harm her health. Due to this the woman takes early postpartum modern contraceptive method from health facility.

P4: This participant said, Yes, there is perception of not get in pregnancy, if she feeds breast milk up to six months.

P5: The participant said, in my opinion some women also belief that modern contraceptive methods dry breast milk, due to this they do not take contraceptive methods early.

**I: Health facility barriers** (service quality, administrative accommodation barriers, providers approach, choices, distance, counseling, IEC, privacy, interaction on family planning during pregnancy, child birth and after birth reminders...)

P1: The participant said, health care providers do not give us method choice of contraceptive methods. **“Example Before I have given this birth, I asked them to give me contraceptive method of injection that can be taken every 3 month, but they said no injection you gave me implants under the skin of my arm which is without my choice. After this I did not take any contraceptive method”.**

P2: There is lack of reminders during and after child birth to the mother to take early postpartum modern contraceptive methods from health care facilities.

P4: This participant said, there is no strong counseling during antenatal care to take early postpartum modern contraceptive methods.

P8: This participant said, health workers educate about breast feed to protect unwanted pregnancy; this may delay to take early postpartum modern contraceptive methods.

P7: The participant said, If the woman needs to take the method health workers deliver the method.

**I: Method-related factors** (Health Concern, accesses, side effects)

P4: The participant said health side effects of contraceptives can be another reason not to use early postpartum modern contraceptive methods.

P7: She reported that, yes, due to the method side effects mothers fear to take early post-partum modern contraceptive.

**I: Cultural barriers** (Probe: encourage high number of children, Social desirablity fear, postpartum practice at home, religious restrictions).

P3: She said that, there is no permission based on religion. It is Haram and considered as killing of human life.

P4: The participant said, home practices after child birth makes busy the woman and goes out of the home before 40 days after child birth is culturally not accept in the community. This can be a reason not to take contraceptive methods early after child birth.

**I: Gender issues** (Probe: Women’s empowerment, male engagement, husband opposition and contraceptive decision making).

P4: She said that, husband opposition due to need of too many children is the reason not to take early postpartum modern contraceptive methods.

P7: The participant said many women want to space child birth, but due to fear of their husbands they take contraceptive method secretly from health facilities.

I: **Financial barriers** (probe: perceived expense of contraception,

P: No

**I: Fertility related factors** (Fertility Preferences, birth spacing, fertility intention...)

P6: The participant said child sex preference can be also a reason to not to uptake early postpartum contraceptive methods. “If the birth outcome is female sex or male sex child consecutively the couples are not interested to uptake early postpartum modern contraceptive methods. They have fertility intention without birth spacing to get the missed male of female sex child and do not use early postpartum contraception.

**I: Misconceptions** (probe: Rumors, secondhand reports of side effects?

P2: The participant said, misconceptions of conception can cause reduction of breast milk can be a barrier to uptake early postpartum contraceptive method uptake.

P5: The participant said, rumors related with contraceptive methods causes infertility is another reason not to uptake early postpartum modern contraceptive methods.

P7: She said, second hand reports like contraceptive methods inserted under the arm can cause difficulty to do job and affects health, makes the body thin and causes bleeding are the common rumors that is barrier to uptake modern early postpartum modern contraceptive methods.

**I: What do you suggest to enhance early postpartum family planning? How?**

P1: The participant reported, need of health education about fertility time after child birth and contraceptive method choices.

P3: She said that, strong counseling during pregnancy related with early postpartum modern contraceptive methods can improve uptake early postpartum modern contraceptive methods.

P5: The participant, early reminders and follow up after child birth also about early postpartum modern contraception.

**I: Thank you! I have finished my questions. Do you have anything to add?**

**P: No, Thank you.**

**I: Thank you very much!**

**End**

**Interviewer impression/comments**

The focus group discussion was good in which the participant response looks open and honest. The participant involved with great interest and their participation level was cooperative. The discussion was completed without any interruption and no any disturbance or noisy happened.
