# Supplementary material for: Barriers and facilitators of early postpartum modern contraceptive method uptake in Dessie and Kombolcha City zones, northeast Ethiopia: Conventional content analysis qualitative study
Source: PLoS One. 2024 Jul 17;19(7):e0305971. doi: 10.1371/journal.pone.0305971 (PMC11253950; doi:10.1371/journal.pone.0305971)
Supplement: S1 Dataset — (ZIP) [file pone.0305971.s001.zip › Supporting information file/IDI_KII and FGD Transcriptions/FGD_Transcription_Segno_3_Niguss Cherie1.docx]

**Exploring barriers/challenges to early postpartum contraceptive method uptake among women**

Region: **Amhara**

Zone/Town: Dessie

Cluster/Sub city: Segno

Location: **North Ethiopia**

Participant category: **Postpartum mothers**

Interviewer name: Niguss Cherie

Transcriber name: Niguss Cherie

Date: 25/11/2022

Start time: 3:30

End time: 4:36

Duration: 66 minutes

**FGD transcriptions of conversions –Segno_NC_3**

| Discussant code | Age | HH condition | Religion | Educational background | Parity | Marital status | Occupation | Monthly income | Residence | Family size | Zone |
| --- | --- | --- | --- | --- | --- | --- | --- | --- | --- | --- | --- |
| P1 | 45 | R | M | No | 3 | M | HW |  | 01 | 3 | Dessie |
| P2 | 30 | O | M | 6 | 2 | M | HW |  | 05 | 4 | Dessie |
| P3 | 25 | O | M | 10 | 3 | M | HW |  | 01 | 5 | Dessie |
| P4 | 23 | R | M | 7 | 1 | M | HW |  | 02 | 3 | Dessie |
| P5 | 30 | R | M | 3 | 4 | M | Gov.t |  | 01 | 6 | Dessie |
| P6 | 35 | R | M | 9 | 5 | M | HW |  | 04 | 9 | Dessie |
| P7 | 25 | O | M | 10 | 3 | M | HW |  | 01 | 5 | Dessie |
| P8 | 23 | R | M | 7 | 1 | M | HW |  | 02 | 3 | Dessie |

**I: Do you heard about early postpartum family planning?**

P: They said not heard before.

**I: When a woman can be pregnant after child birth?**

P1, 3, 4: These participants said the woman can get pregnant after 45 days of child birth.

P2, 5, 6, 8: They said pregnancy can happen after 1 year of child birth.

**I: What is the ideal time to get pregnant to a woman after child birth?**

P2, 3, 5, 6: The participants said the ideal time to get pregnant is 4 years after child birth.

P1, 4, 7, 8: Participants reported that the appropriate time to get pregnant is 5 years after child birth.

**I: How do you comment birth spacing in your communiy?**

P2, 3, 5, 7: They said now days no body needs to give birth with short intervals, but unwanted pregnancy happen due to different reasons like not access methods, knowledge gaps time to pregnancy happen…

**I: What is your role in early postpartum family planning? (Probe :)**

**I: Do you discuss family planning with your partner/ spouse?**

P1, 3: They said, yes, life is together, we discussed with our husbands and plan together.

P2, 4, 5, 6, and 7: These participants said, males are not responsible to family planning and contraceptive methods.

**I: What are your views concerning family planning in general?**

P: The participants said, family planning is important to the health of the mother. Now days no body want to give birth many children.

**I: How comfortable are you to use family planning?**

P: All participants agreed they are comfortable to contraceptive methods and willing to use gain, even if they challenged by health facilities to get method choice.

**I: Is there a particular method you are currently using? Any challenges you have experienced in using it?)**

P3: The participant said, I used the method now taken from private clinic. I faced challenge to get this method, because of when I come to the health center to take the method, the health worker said there is no method here due to expired and no choice. Then I go to private clinic and pay 100 birr to the method which was injected on my arm every 3 month.

**I: Would you please mention facilitating factors (if any) to uptake early postpartum family planning?**

P3, 5, 6: The participants said, to improve uptake of early postpartum modern contraceptive methods there is need of male participation and responsibility, availability of method choice at health facilities and family support.

**I: Would you please explain challenges and barriers encountered to early postpartum family planning? Probe**

**I: Knowledge** (Probe: when pregnancy can happen? birth spacing? methods? where to get the service?)

P3: Women have also lacks knowledge on availability of the method that can be taken within 42 days after child birth at health facilities.

P4: Lack of information about the choices of methods which are comfortable to breast feeding mother.

P6: There is gap on information when to take birth control method after child birth.

P5: Not knowing the time when pregnancy can happen after child birth is the reason not taking early postpartum modern contraceptive method.

P7: If I do not saw monthly bleeding/ menstruation after child birth, mothers think pregnancy not happen. When the woman waits her monthly bleeding, sometimes she gets in unwanted pregnancy.

P8: If the woman breast feed, mothers believe no pregnancy occurs within 6 months. This the main reason to not taking early postpartum modern contraceptive method.

**I: Challenges related to family** (Probe: work load, family support…)

P2: The participant said, I faced challenge to take the method from health facility due lack of family support, for example, I delivered twins and face difficulty to care children when I want to go to health facility to take the method. My husband mother was not cooperative to support and care the children, then I request other person to help me and take the method to prevent unwanted pregnancy.

**I: Attitude** (probe: opposing, method suitablity, Perceived low fecund ability)

P5: The participant said, in my opinion some women also belief that modern contraceptive methods dry breast milk, due to this they do not take contraceptive methods early.

P4: She explained that, if the woman delivers by delivery by C/S, health workers counsel strongly to fear narrow pregnancy that may harm her health. Due to this the woman takes early postpartum modern contraceptive method from health facility

P7: The participant said, there is perception of not get in pregnancy, if she feeds breast milk up to six months.

**I: Health facility barriers** (service quality, administrative accommodation barriers, providers approach, choices, distance, counseling, IEC, privacy, interaction on family planning during pregnancy, child birth and after birth reminders...)

P3: She said, **“I faced challenge to take the method from the health facility. When I went to the health facility the provider said the method was expired and no method choice”.**

P3: The participant said, the health facility service delivery and opening day is not convenient to service users. When I went to the health facility on Saturday morning, the health worker said family planning service was not delivered today due to weekend; no professionals working in family planning unite.

P2: The participant said, if the woman needs to take the method health workers deliver the method.

P3: She reported that, Health workers educate about breast feed to protect unwanted pregnancy; this may delay to take early postpartum modern contraceptive methods.

P4: The participant said, there is no strong counseling during antenatal care to take early postpartum modern contraceptive methods.

P6: There is lack of reminders during and after child birth to the mother to take early postpartum modern contraceptive methods from health care facilities

**I: Method-related factors** (Health Concern, accesses, side effects)

P1: The participant said health side effects of contraceptives can be the reason not to use early postpartum modern contraceptive methods.

P3: She reported that, yes, due to the method side effects like excess bleeding mothers fear to take early post-partum modern contraceptive.

**I: Cultural barriers** (Probe: encourage high number of children, Social desirablity fear, postpartum practice at home,)

P: The participants said, Now day’s no more cultural barriers, it is based on our economy.

**I: Gender issues** (Probe: Women’s empowerment, male engagement, husband opposition and contraceptive decision making)

P4: The participant said, males are not responsible and participatory to family planning. For example “the recent neighbor to me was delivered before a year, now she gave birth again after a year. Due to this her husband blamed her, why you get pregnant with this short birth interval. Then they divorced due to this unwanted pregnancy and she cares the babies now with the small payment of washing cloth of other people”.

P6, 7, 8: They said that, male participation and responsibility on family planning is low and they consider as the only responsibility of the woman.

I: **Financial barriers** (probe: perceived expense of contraception,

R: Financial challenge,

**I: Fertility related factors** (Fertility Preferences, birth spacing, fertility intention...)

Participants: They said, now days this is not barrier.

**I: Misconceptions** (probe: Rumors, secondhand reports of side effects?

P3: Misconceptions of conception can cause reduction of breast milk can be a barrier to uptake early postpartum contraceptive method uptake.

P5: Rumors related with contraceptive methods causes infertility is another reason not to uptake early postpartum modern contraceptive methods.

P7: Second hand reports like contraceptive methods inserted under the arm can cause difficulty to do job and affects health, makes the body thin and causes bleeding are the common rumors that is barrier to uptake modern early postpartum modern contraceptive methods.

**I: What do you suggest to enhance early postpartum family planning? How?**

P3: The participant reported, need of health education about fertility time after child birth and contraceptive method choices.

P5: She said that, strong counseling during pregnancy related with early postpartum modern contraceptive methods can improve uptake early postpartum modern contraceptive methods.

P8: The participant, early reminders and follow up after child birth also about early postpartum modern contraception.

I: Thank you! I have finished my questions. Do you have anything to add?

**R:**

**I: Thank you very much!**

**End**

**Interviewer impression/comments**

The in-depth interview of this key informant was good in which the participant response looks open and honest. The participant involved with great interest and his participation level was cooperative. The interview/discussion was completed without any interruption and no any disturbance or noisy happened. In-depth interview was conducted in separate place in home area after work hour during rest time of key informant.
