# Supplementary material for: Barriers and facilitators of early postpartum modern contraceptive method uptake in Dessie and Kombolcha City zones, northeast Ethiopia: Conventional content analysis qualitative study
Source: PLoS One. 2024 Jul 17;19(7):e0305971. doi: 10.1371/journal.pone.0305971 (PMC11253950; doi:10.1371/journal.pone.0305971)
Supplement: S1 Dataset — (ZIP) [file pone.0305971.s001.zip › Supporting information file/IDI_KII and FGD Transcriptions/KII_Transcription_BW_01_Niguss Cherie.docx]

**Exploring barriers/challenges to early postpartum modern contraceptive method uptake**

Region: **Amhara**

Zone: South Wollo

District/town: Dessie

Location: **North Ethiopia**

Respondent age: 30

Sex: Male

Kebele: 10

Marital status: married

Family size: 3

Religion: Orthodox

HH condition: rent

Occupation: Private organization

Education level: Secondary education level

Participant category: **Husband**

Interviewer name: Niguss Cherie

Transcriber name: Niguss Cherie

Date: 22/11/2022

Start time: 5:00

End time: 5:45

Duration: 45 minutes

**KII transcriptions of conversions –Buanbawuha_NC_01**

I: Do you heard about early postpartum family planning?

R: The respondent said, I, heard about family planning, but did not hear about early postpartum family planning.

I: When a woman can be pregnant after child birth?

R: According to the respondent, he said based on previous cultural thoughts the woman can be pregnant after 6 months of child birth.

I: What is the ideal time to get pregnant to a woman after child birth?

R: The participant said the good time to be pregnant after child birth should be minimum of 2 years.

I: How do you comment birth spacing in your communiy?

R: Base of the respondent information, now day’s people have information about family planning methods. But, sometimes I saw women give birth with short interval.

I: What is your role in early postpartum family planning? (**Probe :**)

I: Do you discuss family planning with your partner/ spouse?

R: The participant said, we did not discuss about family planning before, because we do not live together. This birth happened without our plan, when the problem happen, she went to her family.

I: What are your views concerning family planning in general?

R: The respondent said, family planning is important to the health of the child and the mother and also important to balance our economy with family size.

I: Would you please explain challenges and barriers encountered to early postpartum family planning? **Probe:**

**I: Knowledge** (Probe: when pregnancy can happen? birth spacing?, methods? where to get the service?)

R: The participant said, knowledge gap on the time of pregnancy happening after child birth many women gets in unwanted pregnancy. This participant reported that, women think that I am breast feeding and no risk pregnancy. The participant said women said, if monthly bleeding/ menstruation not seen after child birth I am no probability of pregnancy. Due to this they did not take early postpartum modern contraceptive methods. Now days no body want to pregnant when breast feed.

**I: Challenges related to family** (Probe: work load, Family support)

R: The participant said, I think this cannot be a problem.

**I: Attitude** (probe: opposing, method suitablity, Perceived low fecund ability)

R: The respondent said, in my opinion some women also belief that modern contraceptive methods dry breast milk, make thinness of the body and due to this they do not take contraceptive methods early

**I: Health facility barriers** (service quality, administrative accommodation barriers, providers approach, choices, distance, counseling, IEC, privacy, interaction on family planning during pregnancy, child birth and after birth reminders...)

R: The respondent said that, there is lack of reminders during and after child birth to the mother to take early postpartum modern contraceptive methods from health care facilities. This participant also said, there are no strong information, education and counseling during antenatal care to take early postpartum modern contraceptive methods.

**I: Method-related factors** (Health Concern, accesses, side effects)

R: The participant said, yes I heard before women said contraceptive methods can cause thinness of the body, due to these women may not take early postpartum modern family planning methods.

**I: Cultural barriers** (Probe: encourage high number of children, Social desirablity fear, postpartum practice at home,)

R: Based on the respondent saying, this is not a problem.

**I: Gender issues** (Probe: Women’s empowerment, male engagement, husband opposition and contraceptive decision making)

R: He said that, I think this is also not a problem.

I: **Financial barriers** (probe: perceived expense of contraception,

R: The participant said that, contraceptive methods are free of cost; this is not a challenge to uptake early postpartum modern contraceptive methods.

**I: Fertility related factors** (Fertility Preferences, birth spacing, fertility intention...)

R: The respondent said, this may not be a problem.

**I: Misconceptions** (probe: Rumors, secondhand reports of side effects?

R: He said that, I did not hear in depth about misconceptions of contraceptive methods.

I: What do you suggest to enhance early postpartum family planning? How?

R: The participant said that, if health workers give information, education and counseling during delivery about family planning all women can take early postpartum modern family planning methods.

I: Thank you! I have finished my questions. Do you have anything to add?

**R: No**

**I: Thank you very much!**

**End**

**Interviewer impression/comments**

The in-depth interview of this key informant was good in which the participant response looks open and honest. The participant involved with great interest and his participation level was cooperative. The interview/discussion was completed without any interruption. In-depth interview was conducted in separate place.
