# Supplementary material for: Barriers and facilitators of early postpartum modern contraceptive method uptake in Dessie and Kombolcha City zones, northeast Ethiopia: Conventional content analysis qualitative study
Source: PLoS One. 2024 Jul 17;19(7):e0305971. doi: 10.1371/journal.pone.0305971 (PMC11253950; doi:10.1371/journal.pone.0305971)
Supplement: S1 Dataset — (ZIP) [file pone.0305971.s001.zip › Supporting information file/IDI_KII and FGD Transcriptions/KII_Transcription_BW_02_Niguss Cherie.docx]

**Exploring barriers/challenges to early postpartum modern contraceptive method uptake**

Region: **Amhara**

Zone: South Wollo

District/town: Dessie

Location: **North Ethiopia**

Respondent age: 40

Sex: Female

Kebele: 10

Marital status: married

Family size: 5

Religion: Orthodox

HH condition: Own

Occupation: Midwifery

Education level: Masters

Participant category: **Health Worker**

Interviewer name: Niguss Cherie

Transcriber name: Niguss Cherie

Date: 23/11/2022

Start time: 5:00

End time: 6:00

Duration: 60 minutes

**Transcriptions of conversions –Buanbawuha_NC_01**

**I: Do you heard about early postpartum family planning?**

**R**: The respondent said, yes I heard.

**I: When a woman can be pregnant after child birth?**

**R:** The participant said pregnancy can happen starting from 6 weeks postpartum.

**I: What is the ideal time to get pregnant to a woman after child birth?**

R: She said that, the ideal time of getting pregnancy is after 2-3 years of the previous birth.

**I: How do you comment birth spacing in your communiy?**

**R:** The participant said, sometimes women in the community think breast feeding can prevent pregnancy, but they come to us with unwanted pregnancy with short interval.

**I: What is your role in early postpartum family planning? (Probe :)**

**I:** Do you discuss family planning with your partner/ spouse?

**R:** The respondent said, sometimes we discuss to space birth.

**I: What are your views concerning family planning in general?**

**R:** She said that, now days the community do not need more children, but due to lack of knowledge and rumors in the community about family planning methods they do not take methods early which leads to unwanted and short interval pregnancy.

**I: How do you feel about your partner/ spouse using family planning?**

R: She said that, he supports me.

**I: How comfortable are you to use family planning**?

R: The respondent said that, methods have their side effects, if one method not comfortable to me, I changed to other method.

I: Is there a particular method you are currently using? Any challenges you have experienced in using it?)

R: She said that, I have used implant last 2 years, No more side effects.

**I: Would you please mention facilitating factors (if any) to uptake early postpartum family planning? What mitigation or containment strategies**

R: The participant said that, education and counseling during antennal care, minimizes waiting time at health facility and service integration at immunization program can facilitate uptake of early postpartum modern contraceptive methods.

I: Would you please explain challenges and barriers encountered to early postpartum family planning? Probe

**I: Knowledge** (Probe: when pregnancy can happen? birth spacing?, methods? where to get the service?)

R: The respondent said that, if the woman breast feed, mothers believe no pregnancy occurs within 6 months. This the main reason to not taking early postpartum modern contraceptive method. If I do not saw monthly bleeding/ menstruation after child birth, mothers think pregnancy not happen. When the woman waits her monthly bleeding and lack of information about the choices of methods which are comfortable to breast feeding mother, she gets in unwanted pregnancy. The respondent said, **client’s lacks knowledge on availability of the method that can be taken within 42 days after child birth at health facilities. We give information during antenatal care, but they did not give attention to family planning information.**

**I: Challenges related to family** (Probe: work load, family support)

R: The respondent said that, when women came to family planning they said to me that, my husband not support me to take birth control methods and come to health facility secretly from husband. This indicates that problem of family support to uptake early postpartum contraceptive method.

**I: Attitude** (probe: opposing, method suitablity, Perceived low fecund ability)

R: The respondent said that, there is perception of not to get in pregnancy, if she feeds breast milk up to six months among women as well the community.

**I: Health facility barriers** (service quality, administrative accommodation barriers, providers approach, choices, distance, counseling, IEC, privacy, interaction on family planning during pregnancy, child birth and after birth reminders...)

**R**: The respondent said that, administrative accommodation barriers like client cad draw procedure, long waiting time to the service at health facility, shortage of method choice, providers approach and counseling gap during antenatal care, lack of follow up, no reminder system after child birth and weak information and education system to early postpartum contraceptive method are the common barriers at health facilities. She said also all methods may not be stock out from the health facility, but sometimes choices may not be available due to shortage of methods. During this time clients were not happy to take the available method.

**I: Method-related factors** (Health Concern, accesses, side effects)

**R**: The participants said that, what I observer from clients related with early postpartum modern contraceptive method, “**mothers fear of injectable to take and they said I gave birth last near and not capable of my health to take the method until the child grows some. They fear side effects and belief that may harm to their health”.**

**I: Cultural barriers** (Probe: encourage high number of children, Social desirablity fear, postpartum practice at home,)

**R:** The participant said that, I think no strong culture difficulty to uptake early postpartum modern contraceptive methods.

**I: Gender issues** (Probe: Women’s empowerment, male engagement, husband opposition and contraceptive decision making)

**R**: The participant said that, some clients told me husbands need to give birth in short interval and male participation to uptake early postpartum contraceptive methods is low. She said also contraceptive method uptake decision making mainly done by males and some husbands are opposing using contraceptive methods. Due to this some women come to health facility to take contraceptive methods secretly and unknown by their husbands.

I: **Financial barriers** (probe: perceived expense of contraception,

R: She said that, services are free of charge in our facility. Cost cannot be a problem to uptake contraceptive methods.

**I: Fertility related factors** (Fertility Preferences, birth spacing, fertility intention...)

R: The participant said that, except some women reported that, their husbands need to have fertility intention with short interval, now days people do not need too more children.

**I: Misconceptions** (probe: Rumors, secondhand reports of side effects?

**R**: The respondent said that, second hand reports like contraceptive methods inserted under the arm can cause difficulty to do job and affects health, makes the body thin and causes bleeding are the common rumors that can be barrier to uptake modern early postpartum modern contraceptive methods in their living community.

**I:** What do you suggest to enhance early postpartum family planning? How?

**R**: The respondent said that, to improve early postpartum contraceptive uptake the health facility should avoid any bureaucracy to service accommodation, need of family planning service integration at different service unites like antenatal care and immunization, Avoid long waiting time to the service at the health facility, strong education and counseling during antenatal care about early postpartum contraception and reminder system and follow up mechanisms after child birth about uptake of early postpartum contraceptive methods to women.

**I:** Thank you! I have finished my questions. Do you have anything to add?

**R:** No, I finished.

**I:** Thank you very much!

**End**

**Interviewer impression/comments**

The in-depth interview of this key informant was good in which the participant response looks open and honest. The participant involved with great interest and his participation level was cooperative. The interview/discussion was completed without any interruption and no any disturbance or noisy happened. In-depth interview was conducted in separate place.
