# Supplementary material for: Barriers and facilitators of early postpartum modern contraceptive method uptake in Dessie and Kombolcha City zones, northeast Ethiopia: Conventional content analysis qualitative study
Source: PLoS One. 2024 Jul 17;19(7):e0305971. doi: 10.1371/journal.pone.0305971 (PMC11253950; doi:10.1371/journal.pone.0305971)
Supplement: S1 Dataset — (ZIP) [file pone.0305971.s001.zip › Supporting information file/IDI_KII and FGD Transcriptions/KII_Transcription_Hotie_03_Niguss Cherie.docx]

**Exploring barriers/challenges to early postpartum modern contraceptive method uptake**

Region: **Amhara**

Zone: South Wollo

District/town: Dessie

Location: **North Ethiopia**

Respondent age: 40

Sex: Female

Kebele: 6

Marital status: married

Family size: 4

Religion: Orthodox

HH condition: Own

Occupation: Nurse

Education level: Diploma

Participant category: **Health Worker**

Interviewer name: Niguss Cherie

Transcriber name: Niguss Cherie

Date: 20/11/2022

Start time: 3:00

End time: 3:55

Duration: 55 minutes

**Transcriptions of conversions –Hotie_NC_03**

**I:** Do you heard about early postpartum family planning?

**R**: The respondent said, yes I know about family planning but not heard about early postpartum modern contraceptive methods.

**I:** When a woman can be pregnant after child birth?

**R**: She said that, many women saw menstruation after a month or after 45 days of child birth. Therefore pregnancy can happen starting from 45 days after child birth.

**I:** What is the ideal time to get pregnant to a woman after child birth?

**R:** The participant said, there is a minimum of 2 years interval to be pregnant again.

**I:** How do you comment birth spacing in your communiy?

**R:** The respondent said that, this is town people need long birth interval 3 to 5 years. This can be due to civilization or economic challenges in this urban area.

**I:** What is your role in early postpartum family planning? (**Probe :**)

**I:** Do you discuss family planning with your partner/ spouse?

**R:** The respondent said that, sometimes we talk when it rose by other issues.

**I:** What are your views concerning family planning in general?

**R:** The participant said that, the methods are important to space birth which is crucial to the health of the mother and balance family with their economy.

**I**: How do you feel about your partner/ spouse using family planning?

**R:** She said, he is cooperative and we talk some times.

**I:** How comfortable are you to use family planning?

R: The participant said, I do not use contraceptive methods, because I am infertile.

I: Is there a particular method you are currently using? Any challenges you have experienced in using it?)

R: The respondent said, no method now I have used.

I: Would you please mention facilitating factors (if any) to uptake early postpartum family planning?

R: The respondent said that, the service is available in health facilities, the period is safe to use the method, and IUCD is safe can be offered during this early postpartum period. She said, the challenge to uptake of early postpartum contraceptive method can be, the women said I used before hormonal contraceptives, now I gave birth and I need rest.

I: Would you please explain challenges and barriers encountered to early postpartum family planning? **Probe:**

**I: Knowledge** (Probe: when pregnancy can happen? birth spacing? methods? where to get the service?)

R: The participant said, knowledge gap on the time of pregnancy happening after child birth many women gets in unwanted pregnancy. She reported that, women think that I am breast feeding and no risk pregnancy. The participant said women said, if monthly bleeding/ menstruation not seen after child birth I am no probability of pregnancy. Due to this they did not take early postpartum modern contraceptive methods.

**I: Challenges related to family** (Prob: work load, Religious restrictions

R: The respondent said that, sometimes lack of support from the family and work load my delay the woman to take early postpartum modern contraceptive method.

**I: Attitude** (probe: opposing, method suitablity, Perceived low fecund ability)

R: The respondent said that, there is perception of not get in pregnancy, if she feeds breast. The participant said, in my opinion some women also belief that modern contraceptive methods weaken the hand and face difficulty to do hard work, dry breast milk, due to this they do not take contraceptive methods early.

**I: Health facility barriers** (service quality, administrative accommodation barriers, providers approach, choices, distance, counseling, IEC, privacy, interaction on family planning during pregnancy, child birth and after birth reminders...)

R: The participant said that, there is lack of reminders during and after child birth to the mother to take early postpartum modern contraceptive methods from health care facilities. She also said, there is no strong education and counseling during antenatal care to take early postpartum modern contraceptive methods.

**I: Method-related factors** (Health Concern, accesses, side effects)

R: The respondent that, clients raised many complaints to the method side effects like implants and IUCD can be disappear from the body, weakness to the hand to do job, hair loss and some said causes infertility specially injectable/depo. This can be barriers not to take early postpartum modern contraceptive method.

**I: Cultural barriers** (Probe: encourage high number of children, Social desirablity fear, postpartum practice at home,)

R: The respondent said that, home practices after child birth makes busy the woman and goes out of the home before early postpartum after child birth is culturally not accept in the community. This can be a reason not to take contraceptive methods early after child birth.

**I: Gender issues** (Probe: Women’s empowerment, male engagement, husband opposition and contraceptive decision making)

R: The respondent said that, many women want to space child birth, but due to fear of their husbands and husbands are decision makers they take contraceptive method secretly from health facilities

I: **Financial barriers** (probe: perceived expense of contraception,

R: She said, this cannot be barrier to uptake early postpartum contraceptive method.

**I: Fertility related factors** (Fertility Preferences, birth spacing, fertility intention...)

R: The respondent said, now day’s people do not need more children due to civilization and economic problem.

**I: Misconceptions** (probe: Rumors, secondhand reports of side effects?

R: The respondent that, clients raised many complaints to the method side effects like implants and IUCD can be disappear from the body, weakness to the hand to do job, hair loss and some said causes infertility specially injectable/depo. This can be barriers not to take early postpartum modern contraceptive method.

I: What do you suggest to enhance early postpartum family planning? How?

R: The respondent said that, strong and continuous information, education and counseling during antenatal care, reminders and follow after child birth to uptake early postpartum modern contraceptive methods can improve service utilization.

I: Thank you! I have finished my questions. Do you have anything to add?

**R:** This is what I know.

**I: Thank you very much!**

**End**

**Interviewer impression/comments**

The in-depth interview of this key informant was good in which the participant response looks open and honest. The participant involved with great interest and his participation level was cooperative. The interview/discussion was completed without any interruption and no any disturbance or noisy happened. In-depth interview was conducted in separate place in working area after work hour during rest time of key informant.
