# Supplementary material for: Barriers and facilitators of early postpartum modern contraceptive method uptake in Dessie and Kombolcha City zones, northeast Ethiopia: Conventional content analysis qualitative study
Source: PLoS One. 2024 Jul 17;19(7):e0305971. doi: 10.1371/journal.pone.0305971 (PMC11253950; doi:10.1371/journal.pone.0305971)
Supplement: S1 Dataset — (ZIP) [file pone.0305971.s001.zip › Supporting information file/IDI_KII and FGD Transcriptions/KII_Transcription_Hotie_04_Niguss Cherie .docx]

**Exploring barriers/challenges to early postpartum modern contraceptive method uptake**

Region: **Amhara**

Zone: South Wollo

District/town: Dessie

Location: **North Ethiopia**

Respondent age: 35

Sex: Male

Kebele: 6

Marital status: married

Family size: 3

Religion: Muslim

HH condition: rent

Occupation: religious teacher

Education level: Secondary school

Participant category: **Religious teacher**

Interviewer name: Niguss Cherie

Transcriber name: Niguss Cherie

Date: 20/11/2022

Start time: 4:20

End time: 5:18

Duration: 58 minutes

**Transcriptions of conversions –Hotie_NC_04**

I: Do you heard about early postpartum family planning?

R: The respondent said, I did not hear about it.

I: When a woman can be pregnant after child birth?

R: The respondent said that, the woman can be pregnant after showing menstruation.

I: What is the ideal time to get pregnant to a woman after child birth?

R: The respondent said that, this issue is not accepted in our religion and it can happen when Allah permits it happen.

I: How do you comment birth spacing in your communiy?

R: The participant explained that, there is economic problem in the community that affects the health of the mother and children.

I: What is your role in early postpartum family planning? (**Probe :**)

I: Do you discuss family planning with your partner/ spouse?

R: The respondent said that, this pregnancy is the 1^st^ to us and not discussed.

I: What are your views concerning family planning in general?

**R**: The participant said that, **“birth control methods are not accepted in my opinion and religious thought. If the woman has no health problem no need to use contraceptive methods. He have to because use of birth control methods is considered as killing the life of human being in Sheria thought. If the pregnancy affects the health of the woman it is possible to use and extend 3-4 years the next pregnancy”.**

**I:** How do you feel about your partner/ spouse using family planning?

**R:** The participant said, I do not recommend using contraceptive method. I want to contribute economically what she needs rather to support use of birth control methods.

**I**: How comfortable are you to use family planning?

**R**: The respondent said, this issue not goes with my religion.

**I:** Is there a particular method you are currently using? Any challenges you have experienced in using it?)

**R:** The respondent said, my wife did not use before.

**I:** Would you please mention facilitating factors (if any) to uptake early postpartum family planning?

**R:** The respondent said that, information education and communication to improve awareness both at health facility and religious organization leaders as well.

**I:** Would you please explain challenges and barriers encountered to early postpartum family planning? **Probe:**

**I: Knowledge** (Probe: when pregnancy can happen? birth spacing? methods? where to get the service?)

R: The respondent said, there is knowledge gap about the importance of contraceptive methods. If the pregnancy affects the health of the woman, there is option to use contraceptive methods based on sharia thought.

**I: Challenges related to family** (Probe: work load, family support)

R: The respondent said, there is need of family support to the woman.

**I: Attitude** (probe: opposing, method suitablity, Perceived low fecund ability)

R: The respondent said, use of birth control method considered as killing the life of human beings which affects uptake of early postpartum contraceptive methods.

**I: Health facility barriers** (service quality, administrative accommodation barriers, providers approach, choices, distance, counseling, IEC, privacy, interaction on family planning during pregnancy, child birth and after birth reminders...)

R: The participants said that, **“health care provider’s capacity and approach to deliver services was disappointing. Once up on the time I go to the nearby health center to get service to my mother, they did not do anything. My mother was at the end of death; they did not provide care or refer to other health facility. They gave only glucose on here hand. Finally we discharge by ourselves and go to hospital, and then her health improved. They do not have skill to provide the service, They do not have also drug choice”.**

**I: Method-related factors** (Health Concern, accesses, side effects)

R: The respondent said, I did not hear about it.

**I: Cultural barriers** (Probe: encourage high number of children, Social desirablity fear, postpartum practice at home, religious restriction)

R: The respondent said, there is religion restriction to take modern contraceptive methods and it can be considered as killing the life of the new life. Our religion/Sheria promotes to have more children.

**I: Gender issues** (Probe: Women’s empowerment, male engagement, husband opposition and contraceptive decision making)

**R**: The respondent said that, some male permits to take the method to their wife, in my opinion I do not support to take the method as I told you before due to my region respect.

**I**: **Financial barriers** (probe: perceived expense of contraception,

**R**: The respondent said that, in my opinion this cannot be a problem to uptake the service.

**I: Fertility related factors** (Fertility Preferences, birth spacing, fertility intention...)

**R**: The respondent said that, **“As I told you before I do not support to take birth control methods, rather to have more children and I want to support to fulfill their needs economically”.**

**I: Misconceptions** (probe: Rumors, secondhand reports of side effects?

**R:** The participant said that, there may be rumors in the community, but you can take if it is important. There is need of knowledge to talk more about it.

**I**: What do you suggest to enhance early postpartum family planning? How?

**R:** The respondent said, there is need of information and Education to improve awareness to the community as well to religious leaders and teachers.

**I**: Thank you! I have finished my questions. Do you have anything to add?

**R:** It is enough to me.

**I:** Thank you very much!

**End**

**Interviewer impression/comments**

The in-depth interview of this key informant was good in which the participant response looks open and honest. The participant involved with great interest and his participation level was cooperative. The interview/discussion was completed without any interruption and no any disturbance or noisy happened. In-depth interview was conducted in separate place in home area after work hour during rest time of key informant.
