# Supplementary material for: Barriers and facilitators of early postpartum modern contraceptive method uptake in Dessie and Kombolcha City zones, northeast Ethiopia: Conventional content analysis qualitative study
Source: PLoS One. 2024 Jul 17;19(7):e0305971. doi: 10.1371/journal.pone.0305971 (PMC11253950; doi:10.1371/journal.pone.0305971)
Supplement: S1 Dataset — (ZIP) [file pone.0305971.s001.zip › Supporting information file/IDI_KII and FGD Transcriptions/KII_Transcription_K02_05_Niguss Cherie.docx]

**Exploring barriers/challenges to early postpartum modern contraceptive method uptake**

Region: **Amhara**

Zone: South Wollo

District/town: Kombolcha 02

Location: **North Ethiopia**

Respondent age: 40

Sex: Female

Kebele: 01

Marital status: married

Family size: 4

Religion: Muslim

HH condition: Own

Occupation: Nurse

Education level: Diploma

Participant category: **Health Worker**

Interviewer name: Niguss Cherie

Transcriber name: Niguss Cherie

Date: 20/11/2022

Start time: 3:00

End time: 3:55

Duration: 55 minutes

**Transcriptions of conversions –Kombolcha 02_NC_05**

I: Do you heard about early postpartum family planning?

R: The participant said, yes I heard.

I: When a woman can be pregnant after child birth?

R: The respondent said that, the woman can be pregnant after menstruation immediately or 45 days after child birth.

I: What is the ideal time to get pregnant to a woman after child birth?

R: The respondent said, the appropriate time to a woman to be pregnant is 2-3 years after child birth.

I: How do you comment birth spacing in your communiy?

R: The respondent said that, they need to space birth, but the community belief that if the woman breast feed the child it prevent pregnancy and come to health with unwanted pregnancy.

I: What is your role in early postpartum family planning? (**Probe :**)

I: Do you discuss family planning with your partner/ spouse?

R: The participant said that, yes we have discussed.

I: What are your views concerning family planning in general?

R: The respondent said that, *“****family planning is important to the health of the mother and the child. But, their side effect is difficult to manage. For example I have two children now and the last child is 15 years old. I used injectable for 3 years after child birth and stopped it after three years. But my fertility did not return last 12 years. I have checked at the infertility center and they said no other problem. I think the problem is side effect of the injectable method”.***

I: How do you feel about your partner/ spouse using family planning?

R: The respondent said that, he is cooperative and no problem.

I: How comfortable are you to use family planning?

R: The respondent said that, I do not recommend injectable to newly married couples. Because it causes delay in fertility and sometimes it causes infertility.

I: Is there a particular method you are currently using? Any challenges you have experienced in using it?)

R: The respondent said that, “***I have two children now and the last child is 15 years old. I used injectable for 3 years after child birth and stopped it after three years. But my fertility did not return last 12 years. I have checked at the infertility center and they said no other problem. I think the problem is side effect of the injectable method”***.

I: Would you please mention facilitating factors (if any) to uptake early postpartum family planning?

R: The respondent said that, immunization to their child is good opportunity and strong counseling and education.

I: Would you please explain challenges and barriers encountered to early postpartum family planning? **Probe:**

**I: Knowledge** (Probe: when pregnancy can happen? birth spacing? methods? where to get the service?)

R: The participant said that, ***“if the woman breast feed, mothers believe no pregnancy occurs within 6 months. She said also, if I do not saw monthly bleeding/ menstruation after child birth, mothers think pregnancy not happen. When the woman waits her monthly bleeding, sometimes she gets in unwanted pregnancy. Lack of information about the choices of methods which are comfortable to breast feeding mother is also a reason not to take early postpartum modern contraceptive methods”.***

**I: Challenges related to family** (Probe: work load, family support)

R: The respondent said, this may not be a challenge.

**I: Attitude** (probe: opposing, method suitablity, Perceived low fecund ability)

R: The respondent said that, the communities believe that, if the woman breast feeding pregnancy cannot happen. Due to this many women came to this facility with unwanted pregnancy.

**I: Health facility barriers** (service quality, administrative accommodation barriers, providers approach, choices, distance, counseling, IEC, privacy, interaction on family planning during pregnancy, child birth and after birth reminders...)

R: The respondent said that, we have all method choices in our health facility. There is no gap related with trained health workers.

**I: Method-related factors** (Health concern, accesses or side effects)

R: The respondent said that, mothers said, I need rest from hormonal methods side effects like face color change/madat, irregular bleeding or hand weakness. Clients focus on injectable.

**I: Cultural barriers** (Probe: encourage high number of children, social desirability fear, postpartum practice at home, religious restriction)

R: The respondent said, some husbands and religious belief encourage having high number of children. ***“Some women come here do not want to discontinue child birth. They said, if we do not challenge about feed and drink stop of child birth is haram”.***

**I: Gender issues** (Probe: Women’s empowerment, male engagement, husband opposition and contraceptive decision making).

R: The respondent said, husbands do not support long acting methods. When the women have bleeding side effects due to implants, I advised the woman to take pills, and mothers said the husband see and oppose to take pills daily. This indicates there are husband opposition and no male participation and responsibility to contraceptive method use decision making.

I: **Financial barriers** (probe: perceived expense of contraception,

R: The respondent said that, I think this challenge is not a problem in contraceptive method uptake.

**I: Fertility related factors** (Fertility Preferences, birth spacing, fertility intention...)

R: The respondent said that, many people need to give more birth if they have more different sex ratio among male and female births. If they birth male sex consecutively they need to add female sex child.

**I: Misconceptions** (probe: Rumors, secondhand reports of side effects?

R: The respondent said, this is a big challenge. Mothers complained about the methods from miss conceptions like IUCD disappear in the abdomen, Implants cause hand weakness, Face color change/Madat and irregular bleeding. The event is rare, but if once happen it diffused in the community and affects service intake.

I: What do you suggest to enhance early postpartum family planning? How?

R: The respondent said, there are opportunities to improve uptake of early postpartum modern contraceptive through strong information, education, counseling during antenatal care, delivery and immunization. There is also follow up and reminder mechanism after child birth to the mother about early postpartum modern contraceptive method.

I: Thank you! I have finished my questions. Do you have anything to add?

**R:** I finished, thank you.

**I:** Thank you very much!

**End**

**Interviewer impression/comments**

The in-depth interview of this key informant was good in which the participant response looks open and honest. The participant involved with great interest and his participation level was cooperative. The interview/discussion was completed without any interruption and no any disturbance or noisy happened. In-depth interview was conducted in separate place after work hour during rest time of key informant.
