# Supplementary material for: Barriers and facilitators of early postpartum modern contraceptive method uptake in Dessie and Kombolcha City zones, northeast Ethiopia: Conventional content analysis qualitative study
Source: PLoS One. 2024 Jul 17;19(7):e0305971. doi: 10.1371/journal.pone.0305971 (PMC11253950; doi:10.1371/journal.pone.0305971)
Supplement: S1 Dataset — (ZIP) [file pone.0305971.s001.zip › Supporting information file/IDI_KII and FGD Transcriptions/KII_Transcription_K03_06_Niguss Cherie.docx]

**Exploring barriers/challenges to early postpartum modern contraceptive method uptake**

Region: **Amhara**

Zone: South Wollo

District/town: Kombolcha

Location: **North Ethiopia**

Respondent age: 33

Sex: Male

Kebele: 07

Marital status: married

Family size: 6

Religion: Muslim

HH condition: Own

Occupation: Farmer

Education level: No formal education

Participant category: **Husband**

Interviewer name: Niguss Cherie

Transcriber name: Niguss Cherie

Date: 18/11/2022

Start time: 4:00

End time: 5:10

Duration: 70 minutes

**Transcriptions of conversions –Kombolcha 03_NC_06**

I: Do you heard about early postpartum family planning?

R: The respondent said that, I did not hear before.

I: When a woman can be pregnant after child birth?

R: The respondent said that, the woman can be pregnant after menstruation. Sometimes the menstruation comes once at spot and women may not saw it and pregnancy can happen starting from the 40 day after child birth.

I: What is the ideal time to get pregnant to a woman after child birth?

R: The respondent said, the appropriate time to get pregnant is 5 years until the child support him/her self.

I: How do you comment birth spacing in your communiy?

R: The participant said, it depends on self and different from individual to individual. Many needs birth spacing and others said if I have no economic problem no need of controlling fertility.

I: What is your role in early postpartum family planning? (**Probe :**)

I: Do you discuss family planning with your partner/ spouse?

R: The respondent said that, yes, we discussed. For example if Allah gives us healthy birth outcome, we discussed to space minimum of 3 years.

I: What are your views concerning family planning in general?

R: Based on the respondent, now day’s life is challenging and having many children without our income affects the growth and health of children.

I: How do you feel about your partner/ spouse using family planning?

R: The participant said no problem in my side now. Previously she to contraceptive method without my willingness and there was conflict and resolved by family discussion.

I: How comfortable are you to use family planning?

R: The participant said, I am comfortable and she used before.

I: Is there a particular method you are currently using? Any challenges you have experienced in using it?)

R: He said that, after this birth she did not used any contraceptive method. .

I: Would you please mention facilitating factors (if any) to uptake early postpartum family planning? What mitigation or containment strategies

R: The participant said that, information and education in the community about birth spacing can improve uptake of early postpartum modern contraceptive method uptake.

I: Would you please explain challenges and barriers encountered to early postpartum family planning? Probe

**I: Knowledge** (Probe: when pregnancy can happen?, birth spacing?, methods? where to get the service?)

R: The respondent said, knowledge gap about when pregnancy can happen leads to unwanted and early pregnancy. Sometimes menstruation may disappear with one spot and the woman may not saw it. That leads to unwanted pregnancy. If the woman takes the contraceptive method early after child birth it has no problem.

**I: Challenges related to family** (Probe: work load, family support)

R: The participant said, this can be no more barriers.

**I: Attitude** (probe: opposing, method suitablity, Perceived low fecund ability)

R: He said that, perception of I have not seen menstruation to be pregnant.

**I: Health facility barriers** (service quality, administrative accommodation barriers, providers approach, choices, distance, counseling, IEC, privacy, interaction on family planning during pregnancy, child birth and after birth reminders...)

R: The respondent said, it is good now, but lack of information and education in the community can be barrier to early uptake of contraception after child birth. He said also health providers did not tell us when and how she will take birth control methods after child birth.

**I: Method-related factors** (Health Concern, accesses, side effects)

R: The respondent said that, they injectable and implants are said not comfortable to me and they take pills and forget to take daily. Due to this unwanted pregnancy happened.

**I: Cultural barriers** (Probe: encourage high number of children, social desirability fear, postpartum practice at home, religious restriction)

R: The respondent said, ***use of contraceptive in general is not permitted by religion. Use of contraceptive methods is believed to be discontinuation of the life of human being which is haram. But now days there is economic challenge to have many children and many people use contraceptive methods***.

**I: Gender issues** (Probe: Women’s empowerment, male engagement, husband opposition and contraceptive decision making)

R: The respondent said that, first I did not support my wife to take birth control methods; she took the method without my willingness and get in conflict. Male willingness and involvement can be barrier to take early postpartum modern contraceptive methods.

I: **Financial barriers** (probe: perceived expense of contraception,

R: He said that, this is not a barrier.

**I: Fertility related factors** (Fertility Preferences, birth spacing, fertility intention...)

R: The respondent said, yes, having high number of children is promoted in our community. I heard someone has 30 children from different wives in other area that inspire us to have many number of children.

**I: Misconceptions** (probe: Rumors, second hand reports of side effects?

R: The respondent said that, there are different misconceptions/complaints about the methods like injectable can cause infertility; implants can cause change of face color/Yitebsal. Due to this challenge or second hand reports of side effects can be barriers to uptake of early postpartum modern contraceptive methods.

I: What do you suggest to enhance early postpartum family planning? How?

R: The respondent said information and education in the community, and counseling in the health facility can improve uptake of birth control methods early after child birth. There is also need to have follow-up and reminder system during and after child birth to uptake birth control methods early to prevent narrow birth interval.

I: Thank you! I have finished my questions. Do you have anything to add?

**R:** I finished, thank you.

**I:** Thank you very much!

**End**

**Interviewer impression/comments**

The in-depth interview of this key informant was good in which the participant response looks open and honest. The participant involved with great interest and his participation level was cooperative. The interview/discussion was completed without any interruption and no any disturbance or noisy happened. In-depth interview was conducted in separate place during rest time of key informant.
