# Supplementary material for: Barriers and facilitators of early postpartum modern contraceptive method uptake in Dessie and Kombolcha City zones, northeast Ethiopia: Conventional content analysis qualitative study
Source: PLoS One. 2024 Jul 17;19(7):e0305971. doi: 10.1371/journal.pone.0305971 (PMC11253950; doi:10.1371/journal.pone.0305971)
Supplement: S1 Dataset — (ZIP) [file pone.0305971.s001.zip › Supporting information file/IDI_KII and FGD Transcriptions/KII_Transcription_K03_07_Niguss Cherie.docx]

**Exploring barriers/challenges to early postpartum modern contraceptive method uptake**

Region: **Amhara**

Zone: South Wollo

District/town: Kombolcha

Location: **North Ethiopia**

Respondent age: 30

Sex: Male

Kebele: 02

Marital status: married

Family size: 8

Religion: Muslim

HH condition: Own

Occupation: Nurse

Education level: diploma

Participant category: **health worker**

Interviewer name: Niguss Cherie

Transcriber name: Niguss Cherie

Date: 18/11/2022

Start time: 4:00

End time: 5:00

Duration: 60 minutes

**Transcriptions of conversions –Kombolcha 03_NC_07**

I: Do you heard about early postpartum family planning?

R: The respondent said, yes I heard as a health professional

I: When a woman can be pregnant after child birth?

R: The respondent said that, pregnancy can occur after menstruation or after 45 days of child birth.

I: What is the ideal time to get pregnant to a woman after child birth?

R: The respondent said, the ideal time to be pregnant after child birth 2-3 years after child birth.

I: How do you comment birth spacing in your communiy?

R: The participant said, it is good in our community.

I: What is your role in early postpartum family planning? (**Probe :**)

I: Do you discuss family planning with your partner/ spouse?

R: The respondent said, no, I am infertile and have problem of conception.

I: What are your views concerning family planning in general?

R: The respondent said that, it is important to control fertility to balance family size with the economy, unless it is difficult.

I: How do you feel about your partner/ spouse using family planning?

R: The respondent said, he is comfortable.

I: How comfortable are you to use family planning?

R: The respondent said, it is good in my opinion.

I: Is there a particular method you are currently using? Any challenges you have experienced in using it?)

R: The participant said that, I did not use any method, due to not able to conceive.

I: Would you please mention facilitating factors (if any) to uptake early postpartum family planning? What mitigation or containment strategies

R: The participant said that, strong education and counseling starting from antenatal care can improve uptake of early postpartum modern contraceptive methods.

I: Would you please explain challenges and barriers encountered to early postpartum family planning? Probe

**I: Knowledge** (Probe: when pregnancy can happen? birth spacing? methods? where to get the service?)

R: The respondent said that, the knowledge gap related to the time of pregnancy occurrence like if the woman breast feeding pregnancy cannot happen, but come to us to safe abortion.

**I: Challenges related to family** (Probe: work load, family support)

R: The respondent said that, sometimes lack of family support to care the child can be a barrier to uptake early postpartum modern contraceptive methods.

**I: Attitude** (probe: opposing, method suitablity, Perceived low fecund ability)

R: The respondent said that, the attitude of if the woman breast feed perception of low fecund ability.

**I: Health facility barriers** (service quality, administrative accommodation barriers, providers approach, choices, distance, counseling, IEC, privacy, interaction on family planning during pregnancy, child birth and after birth reminders...)

R: The respondent said, after the conflict crisis we have shortage of contraceptive method logistic problem that affects method choice.

**I: Method-related factors** (Health Concern, accesses, side effects)

R: The participant said that, there are side effects from the methods, but women take the methods.

**I: Cultural barriers** (Probe: encourage high number of children, social desirablity fear, postpartum practice at home, religious restriction).

R: The participant said that, this cannot be problem.

**I: Gender issues** (Probe: Women’s empowerment, male engagement, husband opposition and contraceptive decision making)

R: The participant said, male opposition and difficulty of decision making is a barrier to uptake early postpartum modern contraceptive methods.

I: **Financial barriers** (probe: perceived expense of contraception,

R: The participant said that, the service is free of charge and this cannot be a barrier to uptake early postpartum modern contraceptive methods.

**I: Fertility related factors** (Fertility Preferences, birth spacing, fertility intention...)

R: The participant said, this is not a problem in our area to uptake to use early postpartum modern contraceptive methods.

**I: Misconceptions** (probe: Rumors, secondhand reports of side effects?

R: The participant said that, misconceptions related contraceptive methods like infertility, IUCD can cause cancer, implant can cause hand weakness can be a barrier to uptake early postpartum modern contraceptive methods.

I: What do you suggest to enhance early postpartum family planning? How?

R: The respondents said that, Information, education and counseling to the community and antenatal care, follow up and reminders after child birth can improve uptake of early postpartum modern contraceptive methods.

I: Thank you! I have finished my questions. Do you have anything to add?

**R:** I finished, thank you.

**I:** Thank you very much!

**End**

**Interviewer impression/comments**

The in-depth interview of this key informant was good in which the participant response looks open and honest. The participant involved with great interest and his participation level was cooperative. The interview/discussion was completed without any interruption and no any disturbance or noisy happened. In-depth interview was conducted in separate place after work hour during rest time of key informant.
