# Supplementary material for: Barriers and facilitators of early postpartum modern contraceptive method uptake in Dessie and Kombolcha City zones, northeast Ethiopia: Conventional content analysis qualitative study
Source: PLoS One. 2024 Jul 17;19(7):e0305971. doi: 10.1371/journal.pone.0305971 (PMC11253950; doi:10.1371/journal.pone.0305971)
Supplement: S1 Dataset — (ZIP) [file pone.0305971.s001.zip › Supporting information file/IDI_KII and FGD Transcriptions/KII_Transcription_K05_08_Niguss Cherie.docx]

**Exploring barriers/challenges to early postpartum modern contraceptive method uptake**

Region: **Amhara**

Zone: South Wollo

District/town: Kombolcha

Location: **North Ethiopia**

Respondent age: 35

Sex: Female

Kebele: 05

Marital status: Unmarried

Family size: 8

Religion: Muslim

HH condition: Family

Occupation: Nurse

Education level: BSc nurse

Participant category: **health worker**

Interviewer name: Niguss Cherie

Transcriber name: Niguss Cherie

Date: 18/11/2022

Start time: 4:00

End time: 5:00

Duration: 60 minutes

**Transcriptions of conversions –Kombolcha 05_NC_08**

**I**: Do you heard about early postpartum family planning?

**R:** The respondent said that, yes, I heard as a health professional.

**I:** When a woman can be pregnant after child birth?

**R:** The participant said, the woman can be pregnant after 45 days of delivery, if she has been sexually active.

**I:** What is the ideal time to get pregnant to a woman after child birth?

**R:** The respondent said that, to the health of the mother and the child there should be a minimum of 2 years birth interval.

**I:** How do you comment birth spacing in your communiy?

**R:** The respondent said that, they have awareness about family planning.

**I:** What is your role in early postpartum family planning? (**Probe :**)

**R:** The respondent said, my role is delivery of health education and counseling about family planning.

**I:** Do you discuss family planning with your partner/ spouse?

**R:** The respondent said that, I have no husband.

**I:** What are your views concerning family planning in general?

**R:** The respondent said that, family planning is important to the health of the mother and the child.

**I:** How do you feel about your partner/ spouse using family planning?

**R:** She said that, I have no partner.

**I:** How comfortable are you to use family planning?

**R:** The respondent said, It is important.

**I:** Is there a particular method you are currently using? Any challenges you have experienced in using it?)

**R:** The respondent said, No used before.

**I:** Would you please mention facilitating factors (if any) to uptake early postpartum family planning? What mitigation or containment strategies

**R:** The respondent said, make the service available and accessible can increase uptake of early postpartum contraceptive method.

**I:** Would you please explain challenges and barriers encountered to early postpartum family planning? Probe

**I: Knowledge** (Probe: when pregnancy can happen? birth spacing? methods? where to get the service?)

**R:** The respondent said that, knowledge gap like do not know contraceptive method can be taken within 42 days after child birth, if the woman Breast feed the child they think pregnancy not occur, Sometimes if the woman use method before pregnancy menstruation may not return with in regular time, but they think not showing menstruation pregnancy cannot occur, do not now when pregnancy happen after child birth. Low acceptance/negligence of counseling about early uptake of contraceptive methods can be knowledge gaps which can affect early post-partum modern contraceptive method uptake.

**I: Challenges related to family** (Probe: work load, family support)

**R:** The respondent said that, contraceptive uptake is considered as the responsibility of the woman and there is lack of family support to uptake early postpartum modern contraceptive method.

**I: Attitude** (probe: opposing, method suitablity, Perceived low fecund ability)

**R:** The respondent said, Perception of low fundability if the woman breast feed and method suitability can affect service uptake early.

**I: Health facility barriers** (service quality, administrative accommodation barriers, providers approach, choices, distance, counseling, IEC, privacy, interaction on family planning during pregnancy, child birth and after birth reminders...)

**R**: The respondent said that, health facility barriers like, some trained health professionals do not deliver the service due to skill gap, Poor counseling during antenatal care about early postpartum modern contraception, providers approach during antenatal care and delivery, lack of follow up and reminders after child birth can be health facility gap to promote mother about uptake of early postpartum modern contraceptive method.

**I: Method-related factors** (Health Concern, accesses, side effects)

**R:** The participant said, fear of side effects like dry of breast milk due to modern contraceptive methods can be a barrier to uptake early.

**I: Cultural barriers** (Probe: encourage high number of children, Social desirablity fear, postpartum practice at home, religious restriction)

R: The respondent said, Due to religious perception especially young mothers do not accept counseling about early uptake of modern contraceptive methods.

**I: Gender issues** (Probe: Women’s empowerment, male engagement, husband opposition and contraceptive decision making).

R: The respondent said that, Women are depended on their husband and husband opposition to take the method can be a challenge to uptake modern contraceptive method early after birth. ***For example from my experience a woman of 35 years old had 9 children came from our catchment area to delivery, I asked her why you have this number of children at this age. She said to me all are female sex and need of male child. After child birth, I asked her husband to take card from the room and he asked me, did the birth sex male or female? I said to him the birth is female sex child. He said to me you can name and take the child and he was seriously sad. This indicates there is big challenge of gender problem which affects uptake of modern contraceptive method early.***

**I**: **Financial barriers** (probe: perceived expense of contraception,

**R:** The respondent said, this may not be a barrier, because they pay a too much many to other medical and laboratory services.

**I: Fertility related factors** (Fertility Preferences, birth spacing, fertility intention...)

R: The respondent said that, it is not a problem to uptake contraceptive methods in this urban area.

**I: Misconceptions** (probe: Rumors, secondhand reports of side effects?

**R**: The respondent said, rumors related with the method like disappear in the body, the woman of my neighbor take the method, but she was pregnant. Due to this some may not take the contraceptive methods early after child birth.

**I:** What do you suggest to enhance early postpartum family planning? How?

**R:** Based on the respondent, counseling, health education, mothers conference, promotion through different media and fulfill the service at the nearby health facility can enhance uptake of early postpartum modern contraceptive method.

I: Thank you! I have finished my questions. Do you have anything to add?

**R:** We listed above.

**I: Thank you very much!**

**End**

**Interviewer impression/comments**

The in-depth interview of this key informant was good in which the participant response looks open and honest. The participant involved with great interest and his participation level was cooperative. The interview/discussion was completed without any interruption and no any disturbance or noisy happened. In-depth interview was conducted in separate place
