# Supplementary material for: Barriers and facilitators of early postpartum modern contraceptive method uptake in Dessie and Kombolcha City zones, northeast Ethiopia: Conventional content analysis qualitative study
Source: PLoS One. 2024 Jul 17;19(7):e0305971. doi: 10.1371/journal.pone.0305971 (PMC11253950; doi:10.1371/journal.pone.0305971)
Supplement: S1 Dataset — (ZIP) [file pone.0305971.s001.zip › Supporting information file/IDI_KII and FGD Transcriptions/KII_Transcription_Segno_09_Niguss Cherie.docx]

**Exploring barriers/challenges to early postpartum modern contraceptive method uptake**

Region: **Amhara**

Zone: South Wollo

District/town: Dessie

Location: **North Ethiopia**

Respondent age: 33

Sex: Male

Kebele: 03

Marital status: married

Family size: 8

Religion: Orthodox

HH condition: rent

Occupation: Merchant

Education level: high school

Participant category: **Husband**

Interviewer name: Niguss Cherie

Transcriber name: Niguss Cherie

Date: 14/11/2022

Start time: 8:00

End time: 8:50

Duration: 50 minutes

**Transcriptions of conversions –Segno Gebeya_NC_09**

**I:** Do you heard about early postpartum family planning?

**R:** The respondent said that, I heard about family planning, I do not know about early postpartum modern family planning.

**I:** When a woman can be pregnant after child birth?

**R:** The respondent said pregnancy can happen starting from 45 days after child birth.

**I:** What is the ideal time to get pregnant to a woman after child birth?

**R:** The respondent said that, to the health of the mother and the child there is need of minimum of 3-4 years birth spacing.

**I:** How do you comment birth spacing in your communiy?

**R:** The respondent said that, I think needs more education about family planning,

**I:** What is your role in early postpartum family planning? (**Probe :**)

**I:** Do you discuss family planning with your partner/ spouse?

**R:** The respondent said, yes we have discussed about birth spacing and family planning, this is my first child, after this we plan to space and will take contraceptive methods.

**I:** What are your views concerning family planning in general?

**R:** The respondent said, it is good to the balance family size based on individual economic status.

**I:** How do you feel about your partner/ spouse using family planning?

**R:** This is my first child and I agree to my partner to plan our family.

**I:** How comfortable are you to use family planning?

**R:** The respondent said, it is good using family planning methods.

**I:** Is there a particular method you are currently using? Any challenges you have experienced in using it?)

**R:** The respondent said, the wife not used before modern contraceptive methods.

**I:** Would you please mention facilitating factors (if any) to uptake early postpartum family planning? What mitigation or containment strategies

**R:** The respondent said, family planning depends on individual economic status and no more obstacles to uptake the service.

**I:** Would you please explain challenges and barriers encountered to early postpartum family planning? Probe

**I: Knowledge** (Probe: when pregnancy can happen?, birth spacing?, methods? where to get the service?)

**R:** The respondent said, knowledge problems like not knowing when pregnancy can happen, when the woman can take contraceptive methods after child birth and what methods can used after child birth are barriers to uptake modern contraceptive methods early after child birth.

**I: Challenges related to family** (Probe: work load, family support)

**R:** Based on the respondent, lack of family support and work load at home can be a challenge to uptake contraceptive methods early after child birth.

**I: Attitude** (probe: opposing, method suitablity, Perceived low fecund ability)

**R:** The respondent said that, perceived of low fecund ability and fear of method said effects can be barriers to uptake contraceptive methods early after child birth.

**I: Health facility barriers** (service quality, administrative accommodation barriers, providers approach, choices, distance, counseling, IEC, privacy, interaction on family planning during pregnancy, child birth and after birth reminders...)

**R:** The respondent said, health facility barriers like far distance of health facility, health workers approach and service delivery problem, mistakes from health workers and lack of follow up and reminders after child birth can affect contraceptive method service uptake early after child birth to prevent narrow and unwanted pregnancy.

**I: Method-related factors** (Health Concern, accesses, side effects)

**R:** The respondent said, contraceptive method said effects like fertility delay after discontinuation of the method and fear of infertility, fear of methods affect health and access challenges can be obstacles to uptake modern contraceptive methods.

**I: Cultural barriers** (Probe: encourage high number of children, Social desirablity fear, postpartum practice at home, religious restriction)

**R:** The respondent said that, use of contraceptive methods is not permitted by religion, but individuals should plan based on their economy. Postpartum home practice culturally not permitted to out from home and family opposition can be cultural barriers to uptake early birth control methods from health facilities.

**I: Gender issues** (Probe: Women’s empowerment, male engagement, husband opposition and contraceptive decision making)

**R:** The respondent said, It is the responsibility of husband in my opinion to plan birth spacing, but some husbands my not support their husband to uptake modern contraceptive methods that consider as the responsibility of the woman own.,

**I:** **Financial barriers** (probe: perceived expense of contraception,

**R:** The participant said that, high cost of implants from private health facilities around the home affects the woman to take implants, but the cost of pills relatively low. Due to this they use pills contraceptive methods. Based on the respondent opinion, cost of contraceptive methods affect method choice to uptake early postpartum modern contraceptive methods.

**I: Fertility related factors** (Fertility Preferences, birth spacing, fertility intention...)

**R:** The participant said that, this may not be a problem.

**I: Misconceptions** (probe: Rumors, secondhand reports of side effects?

**R:** The participant said, rumors about side effects of modern contraceptive methods from others affect service uptake early postpartum, but need of appropriate information from the health professionals.

**I:** What do you suggest to enhance early postpartum family planning? How?

**R:** The respondent said, work at the community lower level, information and education about contraceptive methods to husbands can improve early postpartum contraceptive methods.

**I:** Thank you! I have finished my questions. Do you have anything to add?

**R: I finished my idea.**

**I: Thank you very much!**

**End**

**Interviewer impression/comments**

The in-depth interview of this key informant was good in which the participant response looks open and honest. The participant involved with great interest and his participation level was cooperative. The interview/discussion was completed without any interruption and no any disturbance or noisy happened. In-depth interview was conducted in separate place after work hour during rest time of key informant.
