# Supplementary material for: Barriers and facilitators of early postpartum modern contraceptive method uptake in Dessie and Kombolcha City zones, northeast Ethiopia: Conventional content analysis qualitative study
Source: PLoS One. 2024 Jul 17;19(7):e0305971. doi: 10.1371/journal.pone.0305971 (PMC11253950; doi:10.1371/journal.pone.0305971)
Supplement: S1 Dataset — (ZIP) [file pone.0305971.s001.zip › Supporting information file/IDI_KII and FGD Transcriptions/KII_Transcription_segno_10_Niguss Cherie.docx]

**Exploring barriers/challenges to early postpartum modern contraceptive method uptake**

Region: **Amhara**

Zone: South Wollo

District/town: Dessie

Location: **North Ethiopia**

Respondent age: 29

Sex: Female

Kebele: 06

Marital status: married

Family size: 4

Religion: Muslim

HH condition: Rent

Occupation: Midwife

Education level: BSc

Participant category: **Health worker**

Interviewer name: Niguss Cherie

Transcriber name: Niguss Cherie

Date: 14/11/2022

Start time: 9:00

End time: 10:10

Duration: 70 minutes

**Transcriptions of conversions –Segno Gebeya_NC_10**

**I**: Do you heard about early postpartum family planning?

**R**: The respondent said, yes I heard and also trained.

**I**: When a woman can be pregnant after child birth?

**R**: The respondent said that, the woman can be pregnant after 6 weeks postpartum if she is sexually active.

**I**: What is the ideal time to get pregnant to a woman after child birth?

**R**: The respondent said, the appropriate time to be pregnant after child birth is minimum of 2 years.

**I:** How do you comment birth spacing in your communiy?

**R:** The respondent said, this area is town, no more narrow birth interval. But, sometimes we face narrow birth interval for example the woman come to the health facility when at the age of the child was 6 months the woman come to use pregnant.

**I:** What is your role in early postpartum family planning? (**Probe :**)

**R:** The respondent said I took training about early postpartum modern contraceptive methods and counsels the mothers to take early postpartum modern contraceptive.

**I:** Do you discuss family planning with your partner/ spouse?

**R:** The respondent said that, yes we talk about family planning and, I took the method within 45 days after child birth.

**I**: What are your views concerning family planning in general?

**R**: The respondent said, family planning has great role to the health and economic development.

**I**: How do you feel about your partner/ spouse using family planning?

**R**: The respondent said, He supports me and no opposition.

**I**: How comfortable are you to use family planning?

**R**: The respondent said, it is ok to me.

**I:** Is there a particular method you are currently using? Any challenges you have experienced in using it?)

**R:** The respondent said, now I am pregnant, but I used before this pregnancy.

**I:** Would you please mention facilitating factors (if any) to uptake early postpartum family planning? What mitigation or containment strategies

**R:** The respondent said that, counseling during antenatal care and delivery to take early within 6 hours after child birth can improve uptake of early postpartum modern contraceptive method.

**I:** Would you please explain challenges and barriers encountered to early postpartum family planning? **Probe**

**I: Knowledge** (Probe: when pregnancy can happen? birth spacing? methods? where to get the service?)

**R:** The respondent said that, knowledge related barriers to early uptake of postpartum modern contraceptive methods can be they said that we do not have sexual intercourse no need to take early, breast feeding prevents pregnancy up to 2 years.

**I: Challenges related to family** (Probe: work load, family support)

**R:** The respondent said that, work load at home and lack of family support to care child can be factors that inhibit up take of modern contraceptive methods early from health facility.

**I: Attitude** (probe: opposing, method suitablity, Perceived low fecund ability)

**R**: The respondent said that, perception of method not suitable to health like it causes hand weakness and IUCD cause vaginal secretion, husband opposing to take the method can be barriers to uptake contraceptive methods early after child birth.

**I: Health facility barriers** (service quality, administrative accommodation barriers, providers approach, choices, distance, counseling, IEC, privacy, interaction on family planning during pregnancy, child birth and after birth reminders...)

**R**: The respondent said, health facility barriers to uptake early postpartum contraceptive methods can be shortage of trained health care providers, service delivery room problem, follow up and reminders problem after child birth.

**I: Method-related factors** (Health Concern, accesses, side effects)

**R**: The respondent said that, method related factors like IUCD withdraw and unplanned pregnancy, fertility delay and fear of infertility.

**I: Cultural barriers** (Probe: encourage high number of children, Social desirablity fear, postpartum practice at home, religion restriction)

**R**: The respondent said that, religiously they think use of contraception considered as abortion, some mothers reply that I need to give birth and have adequate economy, no need of use contraceptive methods.

**I: Gender issues** (Probe: Women’s empowerment, male engagement, husband opposition and contraceptive decision making)

**R**: The respondent said that, husband opposition to uptake contraceptive methods, no male engagement in family planning service delivery, even sometimes when unwanted pregnancy happen, males consider as the responsibility of the woman to plan the pregnancy and conflict happen with husband. For example I know that the woman have been taken implant in the morning and she came to the health facility afternoon to removal, due to husband disapproval.

**I**: **Financial barriers** (probe: perceived expense of contraception,

**R**: The respondent said, they know it is free.

**I: Fertility related factors** (Fertility Preferences, birth spacing, fertility intention...)

**R**: The respondent said that, fertility preference to have large number of children also can be barrier to uptake of early postpartum modern contraceptive methods.

**I: Misconceptions** (probe: Rumors, secondhand reports of side effects?

**R**: The participant said that, rumors of fertility delay and infertility due to contraception, new clients said fear hand weakness to do day to day tasks due to the implant effect, Knee weakness, excess bleeding, sometimes IUCD withdraw and unwanted pregnancy, they said what I need give birth enough number of children and better use permanent methods are barriers of misconception to uptake early postpartum modern contraceptive methods.

**I:** What do you suggest to enhance early postpartum family planning? How?

**R**: The key informant said that, strong antenatal care counseling about option of early postpartum modern contraceptive methods, reminder and alarm system after child birth to uptake contraceptive methods early after child birth, strong follow up during immunization to take contraceptive methods early, family planning service integration and linkage can improve uptake of early postpartum contraceptive method.

**I:** Thank you! I have finished my questions. Do you have anything to add?

**R:** There is need of information and training to other health professionals.

**I: Thank you very much!**

**End**

**Interviewer impression/comments**

The in-depth interview of this key informant was good in which the participant response looks open and honest. The participant involved with great interest and his participation level was cooperative. The interview/discussion was completed without any interruption and no any disturbance or noisy happened. In-depth interview was conducted in separate place after work hour during rest time of key informant.
